# Modular Lipoprotein Toxins Transferred by Outer Membrane Exchange Target Discrete Cell Entry Pathways

**DOI:** 10.1128/mBio.02388-21

**Published:** 2021-09-14

**Authors:** Christopher N. Vassallo, Govind Prasad Sah, Michael L. Weltzer, Daniel Wall

**Affiliations:** a Department of Molecular Biology, University of Wyoming, Laramie, Wyoming, USA; Brigham and Women’s Hospital/Harvard Medical School

**Keywords:** polymorphic toxins, outer membrane exchange, myxobacteria, kin discrimination, antagonism, lipoproteins

## Abstract

Bacteria compete against related individuals by delivering toxins. In myxobacteria, a key delivery and kin discrimination mechanism is called outer membrane (OM) exchange (OME). Here, cells that display compatible polymorphic cell surface receptors recognize one another and bidirectionally transfer OM content. Included in the cargo is a suite of polymorphic SitA lipoprotein toxins. Consequently, OME between compatible cells that are not clonemates results in intoxication, while exchange between clonemates is harmonious because cells express a cognate repertoire of immunity proteins, which themselves are not transferred. SitA toxins belong to six nonhomologous families classified by sequence conservation within their N-terminal “escort domains” (EDs), while their C termini contain polymorphic nucleases that target the cytoplasmic compartment. To investigate how toxins delivered to the OM by OME translocate to the cytoplasm, we selected transposon mutants resistant to each family. Our screens identified eight genes that conferred resistance in a SitA family-specific manner. Most of these genes are predicted to localize to the cell envelope, and some resemble proteins that colicins exploit to gain cell entry. By constructing functional chimeric SitAs between families, we show that the ED determines the specificity of resistance. Importantly, a mutant that confers resistance to all six SitA families was discovered. This gene was named *traC* and plays an accessory role with *traAB* in OME. This work thus provides insight into the mechanism of kin discrimination in myxobacteria and provides working models for how SitA toxins exploit host proteins to gain cytoplasmic entry.

## INTRODUCTION

Microbes that share similar genotypes and, hence, traits fiercely compete for resources (1, 2). Therefore, the ability to recognize and specifically discriminate against those with common abilities provides strong fitness benefits (3–5). In this evolutionary struggle, bacteria frequently employ toxins or bacteriocins to discriminate (6). One well-known class are the colicins, deployed by Escherichia coli, which target and kill other E. coli cells (2). Another class, which is the focus of this study, are the SitA toxins produced by myxobacteria.

**FIG 1:**
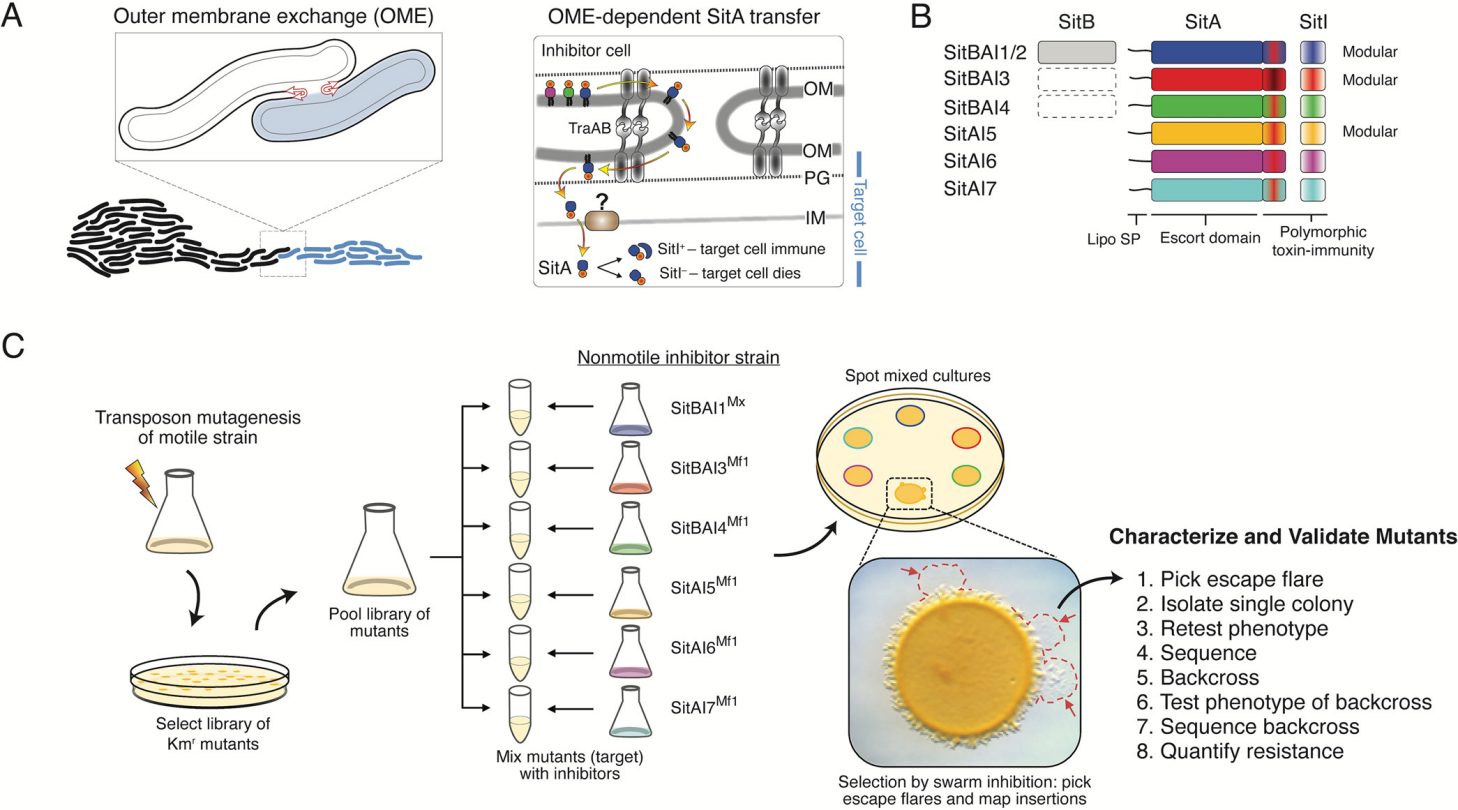
Model of OME and screening strategy. (A, left) Two myxobacterial swarms initiate contact and OME. (Right) Detailed model of TraAB-mediated OM fusion and SitA lipoprotein translocation. PG, peptidoglycan. (B) Schematic of the six SitAI families. Some families contain the SitB accessory protein, which is encoded immediately upstream of *sitA* genes (12, 14). The dashed rectangle indicates that *sitB* is occasionally associated with *sitAI* loci. (C) Screen schematic. The *traAB* merodiploid motile strain was mutagenized to create transposon libraries that were pooled, split, and mixed with the indicated nonmotile inhibitor strains with unique *sitAI* loci. SitA-resistant mutants were identified as escape flares (red arrows) and analyzed. Four to 15 rounds of screening were conducted against each inhibitor strain (see Fig. S1 in the supplemental material).

Myxobacteria are unusually social for microbes, and they use kin recognition to assemble multicellular collectives. Recognition occurs by cell-cell contacts mediated by the highly polymorphic cell surface receptor called TraA and its coreceptor TraB (7–11). Following homotypic cell-cell receptor binding, cells bidirectionally transfer copious amounts of outer membrane (OM) proteins and lipids. This process, called OM exchange (OME), is likely mediated by OM fusion (Fig. 1A). Included as the cargo is a suite of SitA polymorphic toxins that discriminate against nonclonal cells that bear compatible TraA receptors (12). In contrast, clonal cells are resistant because they contain cognate SitI immunity proteins that are not transferred. *sitAI* loci are always found together in an operon and frequently reside on mobile DNA elements (13). The SitA toxins are lipoproteins that belong to six discrete families (SitA1/2, SitA3, SitA4, SitA5, SitA6, and SitA7), which are not homologous to each other but share a domain architecture and TraAB dependence for delivery (Fig. 1B) (14). Strikingly, myxobacterial genomes contain multiple alleles of each family, where total numbers of *sitAI* loci typically range from 25 to 80 per genome. Therefore, the possible numbers of genomic combinations of *sitAI* loci are astronomically high where they serve as precision “self-identity barcodes” that determine social compatibilities between wild Myxococcus xanthus isolates (7, 13, 14).

10.1128/mBio.02388-21.1 FIG S1 Overview of the screening campaign. White boxes represent inhibitor strains (left) used in particular screening rounds, while gray boxes represent round omissions. Each round typically contained pools of ∼2 × 10^4^ independent transposon mutants, except round 8 (*), which contained ∼3 × 10^4^ mutants. Locus tags indicate the rounds in which mutants were isolated. Download FIG S1, TIF file, 0.6 MB. Copyright © 2021 Vassallo et al. 2021 Vassallo et al. https://creativecommons.org/licenses/by/4.0/ This content is distributed under the terms of the Creative Commons Attribution 4.0 International license.

Bacterial systems that deploy toxins, such as OME, face the challenge of delivering the toxin warhead to the appropriate target cellular compartment (15). In some cases, such as the type IV secretion system and the type VI secretion system (T6SS), the toxin can be injected directly into the target cell (16, 17). However, for most systems, including soluble bacteriocins, contact-dependent inhibition (CDI), the type VII secretion system, and even the T6SS, toxins must exploit host factors to translocate across one or two membranes to reach their target compartment (15, 18–25). Although many proteinaceous toxins are active against the cell wall peptidoglycan or cell membranes, nucleases are the most common (26). For colicins, target cell entry is obtained by coopting host machineries, particularly TolA, TolB, TolQ, TolR, or TonB and ExbB and ExbD, which are homologous protein complexes that span the cell envelope. Similarly, CDI toxins contain different translocation domains that hijack different inner membrane (IM) host proteins for cytoplasmic entry (18, 27, 28). In the case of SitA toxins, following OME and delivery to the inner leaflet of the OM, the C-terminal toxins must, through secondary and unknown pathways, traverse the periplasm and IM to gain access to the cytoplasm, where they act as nucleases (Fig. 1A). In this context, we discovered the six SitA families each contain a unique ∼375-amino-acid (aa) conserved N-terminal sequence, termed the “escort domain” (ED), which defines each family (Fig. 1B) (14). Following the EDs are the C-terminal toxin domains, which are polymorphic and, in the case of the SitA1/2, SitA3, and SitA5 families, are also modular. In total, for the six SitA families, 40 distinct toxin domains were identified, most of which are also found in other bacterial toxin systems (14). Given their modular organization, their functions as nucleases and precedence from other toxin systems for cell entry, led to our hypothesis that each ED serves as a translocation vehicle that exploits different host proteins to enter the cytoplasm.

To test our hypothesis and to better understand the mechanism of OME and antagonism, we screened transposon libraries for mutants resistant to SitA toxins. In total, six parallel screens were conducted against each of the SitA families. These screens identified eight genes whose mutations conferred resistance to only the SitA toxin family that they were selected against. Of these, five are predicted proteins that localize to the cell envelope and thus represent candidate cytoplasmic entry pathways. By constructing chimeric SitA proteins and testing their resistance profiles, we indeed found that the EDs determine the specificity of sensitivity and, hence, apparent entry pathways. Importantly, mutations were also discovered in a ninth gene that confers broad resistance to all SitA toxins, and our work indicates that it functions with TraAB in OME.

## RESULTS

### Genetic screen.

Previously, we conducted a screen to identify genetic determinants, besides *traAB*, involved in OME (29). This screen leveraged the powerful phenotype of swarm inhibition (Fig. 1C). This phenotype arises when a motile target (sensitive) strain is mixed with a nonmotile inhibitor strain expressing a unique toxin on agar plates. Antagonism from the inhibitor prevents outward swarming of the motile target strain because it lacks immunity to a particular swarm inhibition toxin (SitA) (14, 30). However, resistant mutants that evade intoxication retain motility and therefore escape swarm inhibition (Fig. 1C). By using this strategy, we screened libraries of transposon mutants in parallel against inhibitor strains that expressed a unique SitA toxin from each of the six families, which were cloned from the divergent strains Myxococcus fulvus (Mf) HW-1 and M. xanthus (Mx) DZ2 (Fig. 1C).

From our previous work, we knew that *traA* or *traB* mutations in the target strain blocked OME and hence were completely resistant to swarm inhibition (29). Consequently, that screen resulted in biased isolation of *traAB* mutations, i.e., 44 of 47 mutants. To minimize this bias, we engineered a second ectopic copy of the *traAB* operon in the wild-type (WT) target M. xanthus DK1622 strain. Although this strategy was successful, a few *traAB* mutants were nevertheless isolated and were disregarded from further analysis. Particular screens contained up to ∼315,000 independent *mariner* transposon mutants. Candidate mutants were isolated as single colonies, backcrossed, and phenotypically verified, and the insertion site was identified (Fig. 1C). These screens identified nine genes that conferred resistance to one or more SitA toxins when mutated (see Fig. S1 in the supplemental material).

### Resistance profiles.

**FIG 2:**
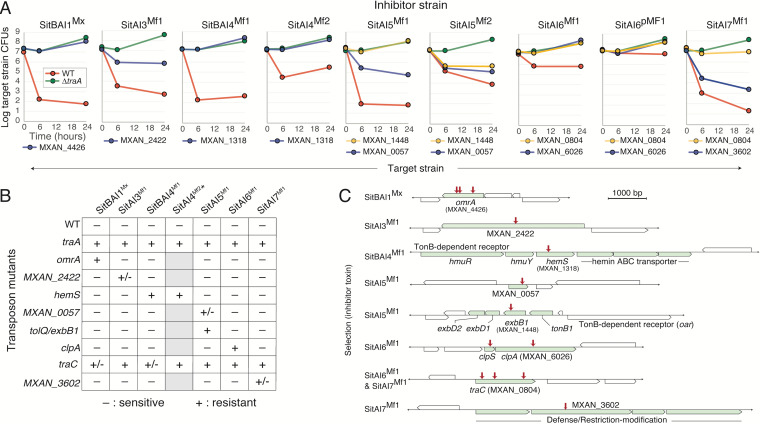
Resistance phenotypes and gene identities of SitA-resistant mutants. (A) Competition assays determined by CFU of backcrossed mutants (Kan^r^) against inhibitor strains expressing the indicated *sitAI* alleles (Kan^s^) at various times. Sensitive (WT) (red lines) and resistant (*traA*::Km^r^) (green lines) controls are shown. Locus tags denote genes with mini-*Himar* insertions. For *sitA4*, -*5*, and -*6*, additional toxin alleles were used to test for cross-resistance within a family. (B) Cross-resistance assessment of inhibitors against target strains. Assays were done as described above for panel A, where a single 24-h time point was measured. −, sensitive; +, resistant (within 1 log of the *traA* mutant at 24 h); +/−, partial resistance (between sensitive and resistant controls). Note that the presence of the *sitB* gene in the indicated inhibitor strains (top) correlated with limited or partial resistance by the *traC* mutant. *This native operon does not contain a *sitB* gene and was not used in the screen (Fig. 1). Gray boxes, not tested. (C) Genes identified from the screen, their neighborhoods, and the inhibitors that they were isolated against. Red arrows represent insertion sites. See Table S1A in the supplemental material for strain details.

Swarm inhibition of motile target cells was caused by cell killing (Movie S1 and Fig. S2). To quantify the degree of resistance, we backcrossed each minitransposon insertion into the WT (target) strain (DK1622) and conducted quantitative killing assays. Here, SitA-producing cells (kanamycin sensitive [Kan^s^]) were competed against Kan-resistant (Kan^r^) WT and Δ*traA* control strains and the test strains, and the CFU of each target strain were determined at 0, 6, and 24 h. As shown, the potency of the SitA inhibitors against the control sensitive strain (WT) varied from ∼10- to 10^7^-fold at the 24-h time point (Fig. 2A). Importantly, all of the backcrossed test strains were resistant, demonstrating that the phenotypes mapped to the transposon insertions. The mutants isolated against SitA1, SitA4, and SitA6 inhibitors exhibited complete resistance, while mutants isolated against SitA3 and SitA7 inhibitors showed partial resistance. In the case of SitA5, partially and fully resistant mutants were isolated in different genes. In conclusion, these results validate the relief of the swarm inhibition phenotype by demonstrating that the isolated mutations conferred resistance to SitA-mediated killing.

10.1128/mBio.02388-21.2 FIG S2 Target strain killing by SitA3^Mx^ toxin delivered by OME. Micrographs are from Movie S1 in the supplemental material. (Left) 0-h time point; (right) 12-h time point. Target cells were labeled with GFP (DW2615) and inhibitor unlabeled (DW2438 expressing the *sitBAI3*^Mx^ locus). The target-to-inhibitor ratio at 0 h was 3:1. Round green spheres in the right panel represent intoxicated cells. Bar, 50 μm. Download FIG S2, TIF file, 1.5 MB. Copyright © 2021 Vassallo et al. 2021 Vassallo et al. https://creativecommons.org/licenses/by/4.0/ This content is distributed under the terms of the Creative Commons Attribution 4.0 International license.

10.1128/mBio.02388-21.9 MOVIE S1 Target cells labeled with GFP (DW2615) were mixed 3:1 with an unlabeled inhibitor (DW2438 expressing the *sitBAI3*^Mx^ locus). Cells were placed on an agarose pad, and micrographs were taken every 30 s for 12 h. Download Movie S1, AVI file, 19.5 MB. Copyright © 2021 Vassallo et al. 2021 Vassallo et al. https://creativecommons.org/licenses/by/4.0/ This content is distributed under the terms of the Creative Commons Attribution 4.0 International license.

10.1128/mBio.02388-21.8 TABLE S1 (A) Strains used in this study; (B) plasmids used in this study; (C) primers used in this study. Download Table S1, DOCX file, 0.04 MB. Copyright © 2021 Vassallo et al. 2021 Vassallo et al. https://creativecommons.org/licenses/by/4.0/ This content is distributed under the terms of the Creative Commons Attribution 4.0 International license.

### SitA resistance genes.

From our previous screen (29), three independent mutants in *omrA* (MXAN_4426) were isolated when selecting against an inhibitor expressing SitBAI1^Mx^ and SitBAI2^Mx^, which belong to the same family. In the initial rounds of this screen (Fig. S1), three independent and fully resistant mutants in *omrA* were also isolated against SitBAI1^Mx^ inhibition (Fig. 2A). We concluded that the SitA1/2 screen reached saturation, and screening against this family was halted after four rounds. Similarly, once mutants that were fully resistant to SitA4 (MXAN_1318) and SitA5 (MXAN_1448) were isolated, screening was stopped (Fig. S1). However, in the case of SitA5, in that same round (ninth), a mutant that conferred partial resistance was also obtained (MXAN_0057) (Fig. 2A and Fig. S1). The eighth screening round identified SitA6-resistant mutants in the ClpA protease (MXAN_6025) and the upstream gene for the ClpS protease adaptor (Fig. 2A and C and Fig. S1), which conferred full resistance. However, since these were well-characterized cytoplasmic proteins that would not be directly involved in toxin entry, we continued screening. Throughout all rounds, a mutant with full resistance to SitA3 or SitA7 was not isolated; however, mutations that conferred partial resistance, MXAN_2422 and MXAN_3602, were found, respectively (Fig. 2A). In the 12th and 15th rounds, a gene (MXAN_0804) was hit three independent times when selecting against SitA6 and SitA7 (Fig. 2A and Fig. S1). Details of these proteins are shown in Fig. S3.

10.1128/mBio.02388-21.3 FIG S3 Overview of proteins identified from screens. Functional predictions derived from HHpred. SP, signal peptide; NT, N-terminus. Download FIG S3, TIF file, 1.7 MB. Copyright © 2021 Vassallo et al. 2021 Vassallo et al. https://creativecommons.org/licenses/by/4.0/ This content is distributed under the terms of the Creative Commons Attribution 4.0 International license.

### Resistant mutants are specific to SitA families.

To address mechanisms and specificity, resistant mutants were tested against a panel of SitA inhibitor strains. As shown, each of the eight mutants was resistant to only the single SitA family that it was isolated against; these mutants showed no cross-resistance (Fig. 2B). In contrast, mutations in MXAN_0804 conferred partial or full resistance to all six SitA families. This gene was named *traC* and is discussed further below. In conclusion, eight of nine mutants conferred resistance in a SitA family-specific manner.

### Cross-resistance occurs within SitA families and is not toxin specific.

One possible mechanism of resistance was to neutralize the lethality of toxins. To address this possibility, from our laboratory collection, we tested alternative alleles of *sitAI1*, *sitAI4*, *sitAI5*, and *sitAI6* against cognate resistance strains. In all four cases, the backcrossed mutants were resistant to alternative alleles within particular SitA families (Fig. 2A and Fig. S4), thus demonstrating that cross-resistance can occur within these SitA families, although it is not absolute (see below). As outlined below, these findings suggest that resistance mutations may be specific to the SitA escort domain and, thus, specific to SitA families.

10.1128/mBio.02388-21.4 FIG S4 OmrA mutation confers cross-resistance against the SitA1/2 family. SitA1^Mx^ contains a colicin-DNase (PF12639) toxin, while SitA2^Mx^ contains a CdiA-CTD DNase toxin. Competitions done as described in the legend of Fig. 2A. Download FIG S4, TIF file, 0.7 MB. Copyright © 2021 Vassallo et al. 2021 Vassallo et al. https://creativecommons.org/licenses/by/4.0/ This content is distributed under the terms of the Creative Commons Attribution 4.0 International license.

Importantly, of the six SitA families, three (SitA4, SitA6, and SitA7) contain toxin domains that are not modular (Fig. 1B). That is, they contain the same toxin domain family, although their alleles vary. In contrast, the toxin domains in the other three families (SitA1/2, SitA3, and SitA5) are polymorphic and modular; combined, they contain at least 37 distinct toxin families (14). For the tested alleles of SitA5^Mf2^, SitA4 (both alleles), and SitA6 (both alleles), they all contain AHH nucleases (Pfam family PF14412), but the toxin modules are not homologous between SitA families. Although these five toxins work by the same presumed mechanism, there was no cross-resistance among these three SitA families (Fig. 2B), suggesting that the corresponding resistance mechanisms do not cause a general block in AHH nuclease activity. Moreover, in the case of SitA5, we tested two alleles where their toxin domains were not homologous: one contained an HNH nuclease (PF13391) (SitA5^Mf1^), and the other contained an AHH nuclease (SitA5^Mf2^). Importantly, both resistant mutants isolated, MXAN_0057 and MXAN_1448, conferred cross-resistance between these two SitA5 toxins (Fig. 2A). Finally, consistent with previous findings (29), OmrA mutants were resistant to both SitA1^Mx^ and SitA2^Mx^ within the SitA1/2 family (Fig. S4), even though they contain different toxin domains, DNase-colicin (PF12639) and CdiA-CTD (C-terminal domain) DNase, respectively. Taken together, these results indicate that most or possibly all of the above-mentioned resistant mutants do not block toxin activity directly but instead work by an alternative mechanism(s).

### SitA toxins are functionally modular.

**FIG 3:**
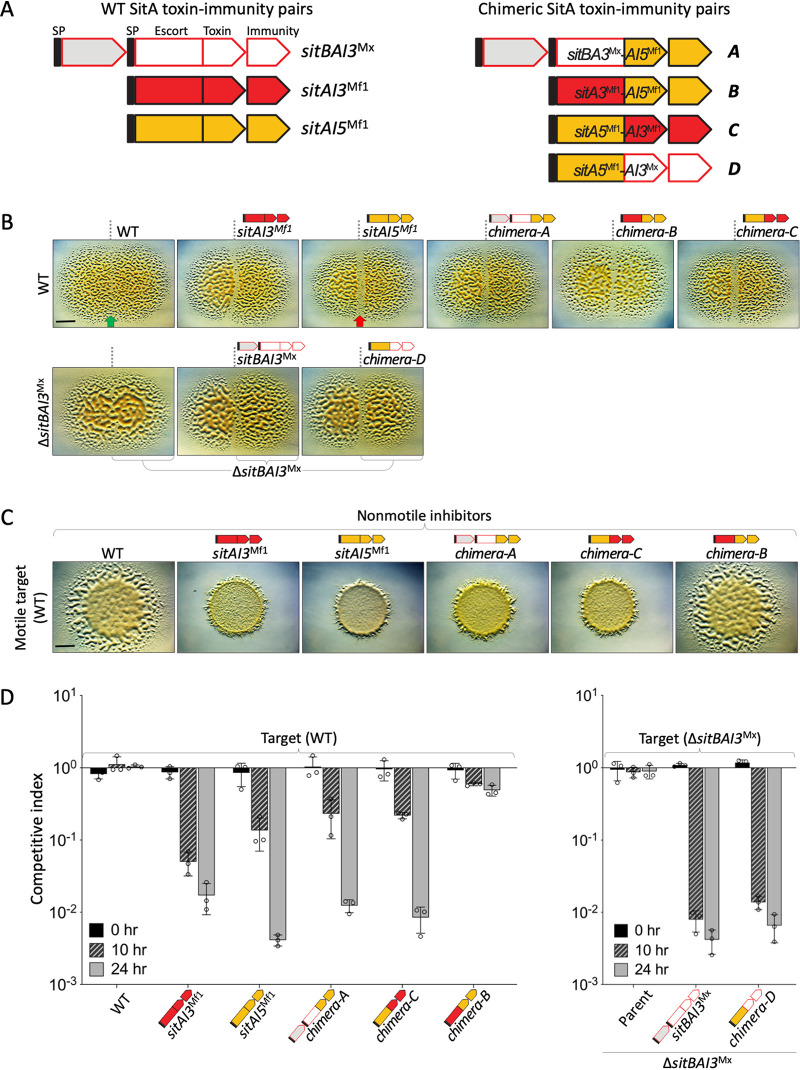
SitA toxins are functionally modular. (A) Schematic of three SitAI proteins used to construct four chimeras. The toxin domains are SitA3^Mx^ Cdi_tRNase (PF18451), SitA3^Mf1^ Tox-RNase-5 (PF15648), and SitA5^Mf1^ HNH nuclease. (B) Colony merger assays where the respective indicator (sensitive) strains (left) were spotted adjacent to test strains expressing the indicated *sitAI* loci (right) and incubated for 2 days. Green arrow, merging colonies; red arrow, demarcation. Bar, 5 mm. (C) Swarm inhibition by chimeras and parent *sitAI* alleles expressed in isogenic nonmotile strains tested against a motile indicator (WT) strain. The left panel shows the no-inhibition control; the next two panels show parent *sitAI* allele controls. Bar, 5 mm. (D) Competition indices of chimeras and parent *sitAI* alleles done with fluorescently labeled target cells (see Fig. S5A in the supplemental material). The ratios of green fluorescent protein (GFP)-labeled target cells (WT) (left) and Δ*sitBAI3*^Mx^ (removes endogenous *sitI3*^Mx^) cells (right) were plotted against unlabeled inhibitor cells (bottom) (note the negative controls on the left). Standard deviations (SDs) from three biological replicates are shown.

Our experimental and bioinformatic analyses indicate that SitA toxins contain modular architecture features. To investigate this hypothesis, we created four reciprocal chimeras (A to D) that swapped escort and toxin domains between the SitAI3 and SitAI5 families and assayed them for activity (Fig. 3A). The first test was a colony merger assay, where a sensitive indicator strain (DK1622 [WT]) swarmed up to a strain expressing a unique *sitAI* chimera cassette. Here, if the latter strain produces a functional toxin, a demarcation appears, while in contrast, clonal colonies do not antagonize and merge seamlessly. In this assay, all four chimeras appeared active (Fig. 3B). The second and more stringent assay was based on swarm inhibition. Here, chimeras A and C were fully active, while chimera B (SitA3^Mf1^-AI5^Mf1^) was not active (Fig. 3C). The latter finding was not contradictory because colony demarcations can be caused by nonkilling mechanisms (31). The fourth chimera, chimera D (SitA5^Mf1^-AI3^Mx^), was not tested because the construction of the required nonmotile parental inhibitor strain (Δ*mglBA* without the endogenous parental allele, Δ*sitAI3*^Mx^) was unsuccessful. Nevertheless, in a third quantitative competition assay, which measures the relative ratios of competing strains over time, chimeras A, C, and D were active at levels near those of the parent allele, while chimera B again showed poor activity (Fig. 3D and Fig. S5A). Based on these combined results, we conclude that the SitA3 and SitA5 families were modular because in three of four cases, their escort and toxin domains were functionally swapped between families.

10.1128/mBio.02388-21.5 FIG S5 Functional SitA chimeras and sequence conservation. (A) Representative examples of competition experiments with chimeras and parent alleles against a target strain (WT) (GFP). Labeled and unlabeled cells were enumerated, and ratios plotted (Fig. 3D). Starting ratios were 1:1. Bar, 5 μm. (B) Sequence alignment of SitA3 proteins by MUSCLE. Download FIG S5, TIFF file, 10.6 MB. Copyright © 2021 Vassallo et al. 2021 Vassallo et al. https://creativecommons.org/licenses/by/4.0/ This content is distributed under the terms of the Creative Commons Attribution 4.0 International license.

### Resistance mechanisms are escort domain specific.

**FIG 4:**
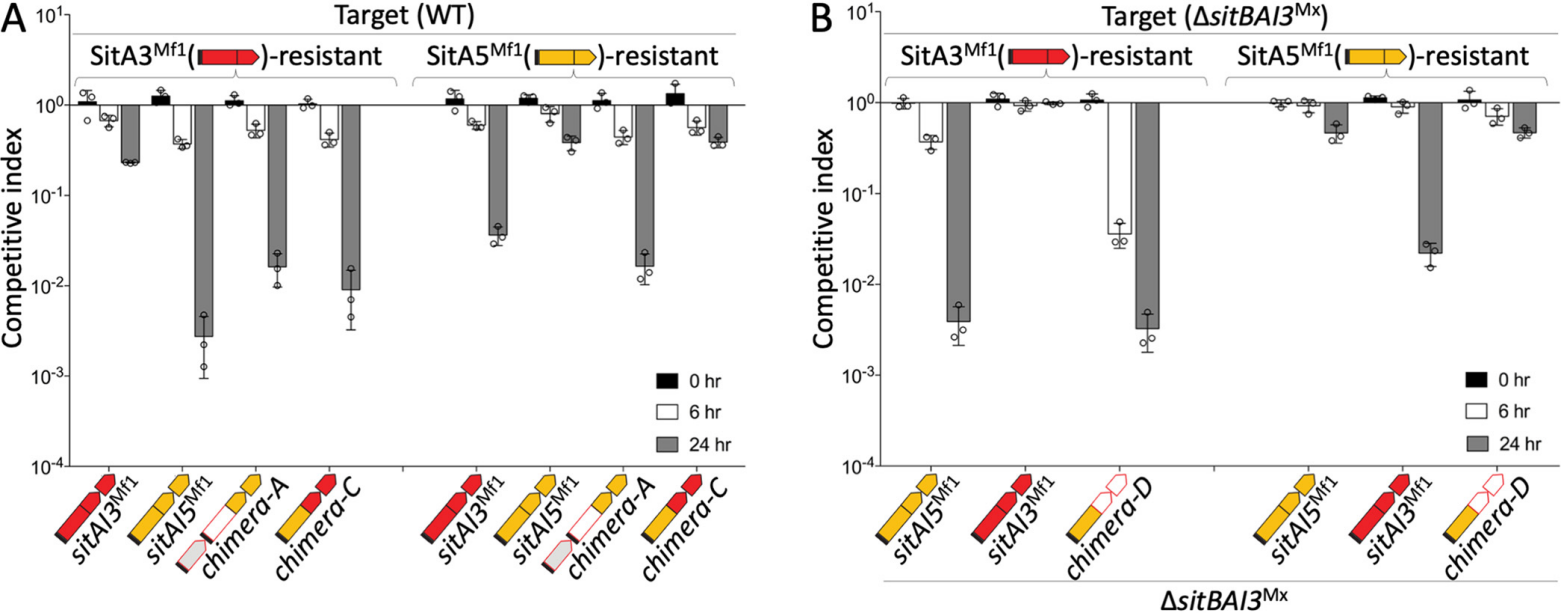
Divergent SitA EDs dictate resistance profiles. (A) Competition indices of chimeras and parent *sit*(*B*)*AI* alleles where target cells were fluorescently labeled (top). The resistance profile of SitA5^Mf1^-AI3^Mf1^ (chimera C) was determined by its ED, while SitBA3^Mx^-AI5^Mf1^ (chimera A) was active against both resistant strains. (B) Competition indices as described above for panel A, except that the target strain was Δ*sitI3*^Mx^ (Δ*sitBAI3*^Mx^), to the test SitA5^Mf1^-AI3^Mx^ (chimera D), where the SitA5^Mf1^ ED determines the resistance profile. SDs from three biological replicates are shown.

Our findings are consistent with the hypothesis that the six SitA families use different cellular pathways to gain cytoplasmic entry, which were blocked by specific resistant mutants. To further investigate this possibility, we tested whether our three functional chimeras exhibited resistance profiles that correlated with their EDs and were independent of the toxin domains. In competition assays, chimeras C and D behaved as predicted; their resistance profiles were determined by their EDs and were independent of their toxin domains (Fig. 4A and B). That is, both of these chimeras with SitA3 toxin domains killed the SitA3-resistant strain (*mxan_2422*::*mariner*), while the SitA5-resistant strain (*exbB1*::*mariner*) exhibited full resistance, which correlated with their SitA5 ED. These results thus support our hypothesis that the resistance phenotypes act on the EDs and, hence, likely block cytoplasmic entry.

**FIG 5:**
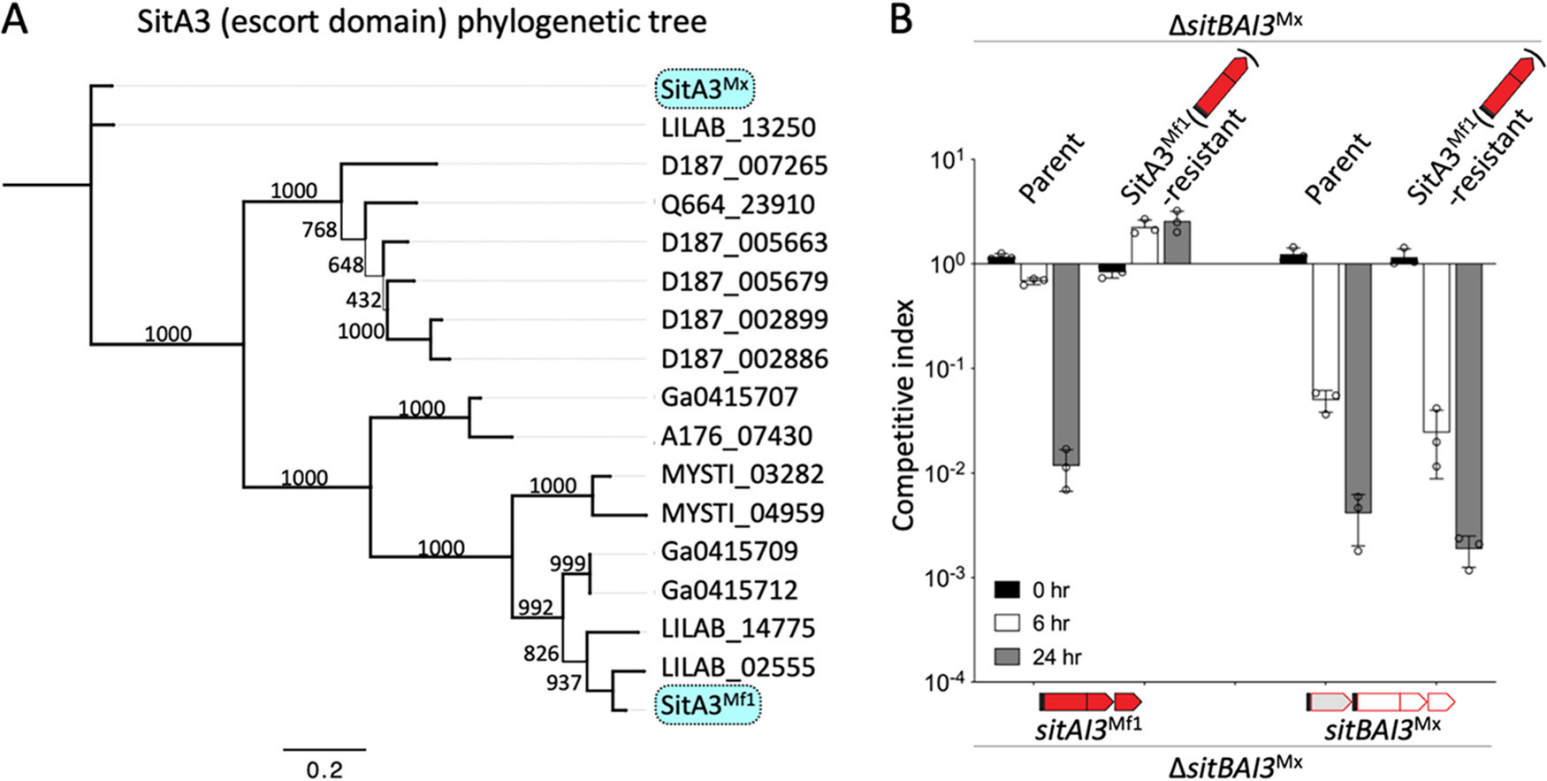
SitA3 EDs are phylogenetically and functionally divergent. (A) Phylogenetic relationships of the SitA3 ED family. Bootstrap values are shown; the bar indicates amino acid substitutions per residue. See Fig. S5B in the supplemental material for a full-length alignment of SitA3^Mx^ versus SitA3^Mf1^. (B) Competition indices of two isogenic inhibitor strains expressing different alleles of *sit*(*B*)*AI3* (bottom) against fluorescently labeled target strains (top). SDs from three biological replicates are shown.

Surprisingly, and in contrast to the above-described findings, chimera A (SitBA3^Mx^-AI5^Mf1^) was active against both the SitA3- and SitA5-resistant mutant strains (Fig. 4A). This puzzling result led us to consider whether EDs within the SitA3 family were divergent in terms of sequence, perhaps explaining why the SitA3 resistance mutation did not affect its toxicity. To address this, we reexamined the sequence conservation among EDs within the SitA3 family. In particular, we compared the SitA3^Mx^ and SitA3^Mf1^ sequences because the *mxan_2422*::*mariner* resistant mutant was isolated against the latter, while chimera A contained an ED from SitA3^Mx^ (Fig. 3). Sequence alignment between these proteins showed that their EDs were only 51.8% identical and contained 7 indel regions over ∼375 aa residues (Fig. S5B). In a broader analysis of 17 SitA3 EDs, the SitA3^Mx^ and SitA3^Mf1^ sequences were actually the most divergent (Fig. 5A). These findings suggested that the function of these EDs may have diverged to recognize distinct host proteins. To test this, the SitA3 resistance allele *mxan_2422*::*mariner* was placed in a Δ*sitBAI3*^Mx^ target strain (to remove natural resistance from *sitI3*^Mx^ present in the laboratory strain). As predicted, this target strain was resistant to SitAI3^Mf1^ but was sensitive to an isogenic strain ectopically expressing SitBAI3^Mx^ (Fig. 5B). We conclude that the sequence divergence within the SitA3 ED family resulted in divergent functions.

### TraC is an accessory factor for OME function.

Besides *traA* and *traB*, the *traC* mutant was the only mutant that conferred resistance to all six SitA families (Fig. 2B). In the case of SitBAI1^Mx^ and SitBAI4^Mf1^, resistance was partial, while the *traA* mutant conferred complete resistance. These two toxin cassettes were the only ones from our panel that contained the *sitB* accessory gene, which we previously showed increases toxin potency by increasing OME transfer efficiency (12). Consistent with this, unlike SitBAI4^Mf1^, the *traC* mutant showed complete resistance to SitAI4^Mf2^, which naturally lacks an upstream *sitB* gene (Fig. 2B). Nevertheless, since the *traC* phenotypes were similar to those of the *traA* and *traB* mutants, we investigated whether TraC functions in OME.

TraC is a predicted 604-aa OM-localized lipoprotein that contains three tetratricopeptide repeats (TPRs) and an uncharacterized C-terminal domain (Fig. S3). TPR motifs are known to mediate protein-protein interactions (32). Although the *traC* locus was not near the *traAB* operon, in comparative analyses, *traC* cooccurred in nearly all myxobacterial genomes that contained bona fide *traAB* and *sitAI* loci (an exception being *Aggregicoccus* sp. strain 17bor-14) and was absent in genomes that did not contain *traAB* and *sitAI* (10, 14), again supporting a role in OME.

**FIG 6:**
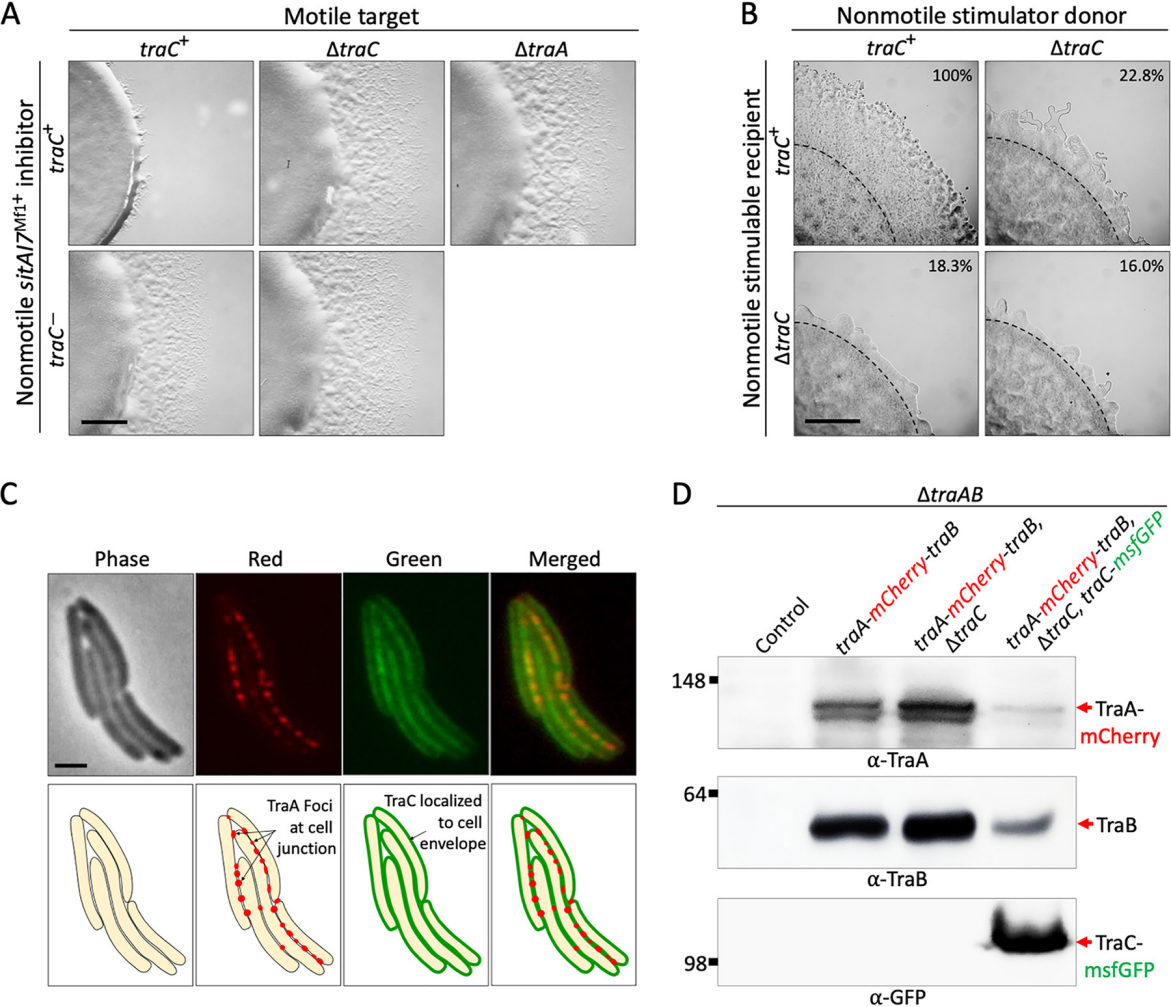
TraC mutants are generally defective in OME. (A) Swarm inhibition of positive (*traC*^+^) and negative (Δ*traA*) controls and *traC* mutants in either the motile target or the nonmotile inhibitor. (B) Rescue of motility defects by cell-cell transfer of motility proteins (OME) between nonmotile strains (stimulation assays). The Δ*traC* mutation results in stimulation (OME) defects in the donor, the recipient (Δ*cglC* Δ*tgl*), or both strains. Stimulation of outward swarms is normalized as a percentage of the positive control (100%). Dashed lines mark the initial inoculum edge. See Fig. S6 in the supplemental material for additional controls. For panels A and B, micrographs were taken at 48 h, and strains were mixed 1:1. Bars, 1 mm. (C) Cell envelope localization of the TraC-msfGFP fusion. Upon cell-cell contact, the TraA-mCherry receptors coalesce at junction sites (foci) (36), while TraC-monomeric superfolder GFP does not. Bar, 2 μm. (D) Representative Western blots of TraA/B/C protein levels from the indicated strains. TraA and TraB levels were elevated 36 and 22% in a Δ*traC* background, respectively, compared to the reference strain (second lane). In contrast, in the TraC overexpression strain (P*_pilA_*-*traC*-*msfGFP*), TraA and TraB levels were reduced 39 and 35%, respectively, compared to the reference strain. The negative-control lane is a Δ*traAB* strain. Molecular weight standards (in kilodaltons) and primary antibodies are shown. See Fig. S7C for the loading control.

OME requires the TraAB machinery to be present in all engaged cells (33). Therefore, to test for a role in OME, we tested whether TraC functions in inhibitor cells. As shown, when a motile target or a nonmotile inhibitor contained a *traC* mutation, SitAI7^Mf1^ swarm inhibition was blocked to similar degrees (Fig. 6A). This result indicates that like TraAB, TraC was required in both cell types to elicit the swarm inhibition phenotype.

10.1128/mBio.02388-21.6 FIG S6 Complementation test for TraC-msfGFP. Stimulations performed between a nonmotile recipient (Δ*tgl* Δ*cglC*) and indicated controls or the test strain. Red arrows mark small flares of stimulated cells. Bar, 1 mm. Download FIG S6, TIFF file, 2.4 MB. Copyright © 2021 Vassallo et al. 2021 Vassallo et al. https://creativecommons.org/licenses/by/4.0/ This content is distributed under the terms of the Creative Commons Attribution 4.0 International license.

10.1128/mBio.02388-21.7 FIG S7 Effect of TraC on TraAB expression. (A) TraA-mCherry foci formation upon cell-cell contact was not impacted by a *traC* mutation. Bar, 2 μm. (B) TraC levels are not impacted by *traA* deletion or overexpression (OE) of TraAB. Western blotting of strains containing FLAG-tagged TraC. Equal amounts of cell material, as determined by culture density, were loaded into each lane. The negative-control strain has no FLAG tag (left), while the remaining strains contained TraC-FLAG in indicated backgrounds. WT *traAB* served as a positive control where the band intensity was arbitrarily set to 1.00. Compared to this control, the band intensity for the Δ*traA* mutant was 0.99, and that for *traAB* overexpression was 0.97. The bottom panel (*) serves as a nonspecific loading control from the same blot that was overexposed. (C) Loading control for Fig. 6D. *, nonspecific cross-reacting band probed with TraA serum. See Table S1A in the supplemental material for strain details. Download FIG S7, TIF file, 1.0 MB. Copyright © 2021 Vassallo et al. 2021 Vassallo et al. https://creativecommons.org/licenses/by/4.0/ This content is distributed under the terms of the Creative Commons Attribution 4.0 International license.

Another test for OME is a “stimulation assay” that involves a mixture of two nonmotile strains. Here, the “donor” has mutations (Δ*mglBA*) in cytoplasmic proteins that permanently block motility, while the “recipient” has mutations in two different lipoproteins, namely, CglC and Tgl, required for adventurous and social motility, respectively. The latter proteins are transferable by OME, and consequently, the recipient can be transiently stimulated for gliding motility from donors that make functional CglC and Tgl (34, 35). When OME is abolished by *traA* or *traB* mutations, no stimulation occurs, and sharp colony edges are produced (Fig. S6) (33). To test for stimulation, we constructed nonmotile donor and recipient strains that contained the Δ*traC* mutation and tested each combination for OME. Importantly, when the Δ*traC* mutation was in the donor, the recipient, or both strains, stimulation was defective but not abolished (Fig. 6B). This sensitive assay highlights the importance of TraC in OME.

Previous screens found that TraA and TraB were the only proteins known to be absolutely required for OME (29). Here, the OME defect of TraC mutants was severe but partial, suggesting that TraC functions as an accessory factor for TraAB. In this context, TraAB localize on the cell surface, and when cells engage in OME, these dynamic receptors coalesce into aggregates or foci, likely representing OM fusion junctions (36). To test for colocalization, a functional TraC-msfGFP reporter was made (Fig. S6). As predicted, TraC-msfGFP lipoprotein localized to the cell envelope; however, upon cell-cell contact, it did not coalesce into aggregates like the TraA-mCherry reporter (Fig. 6C). Additionally, in a Δ*traC* background, TraA-mCherry still formed foci upon cell-cell contacts, consistent with a basal level of OME (Fig. S7A). Next, we assessed the relative levels of TraAB in strains that lacked TraC. Strikingly, in a Δ*traC* background, the TraA and TraB protein levels were elevated relative to the *traC*^+^ control strain (Fig. 6D). In a complementary approach, when TraC was overexpressed from the chromosome by the heterologous *pilA* promoter, TraA and TraB protein levels were reduced (Fig. 6D). In contrast, in a Δ*traA* or *traAB* overexpression backgrounds, TraC levels remained unchanged compared to the *traAB* WT control (Fig. S7B). Based on these combined findings, we conclude that TraC plays an accessory role in OME, perhaps by regulating the function, processing, and/or turnover of the TraAB proteins.

## DISCUSSION

**FIG 7:**
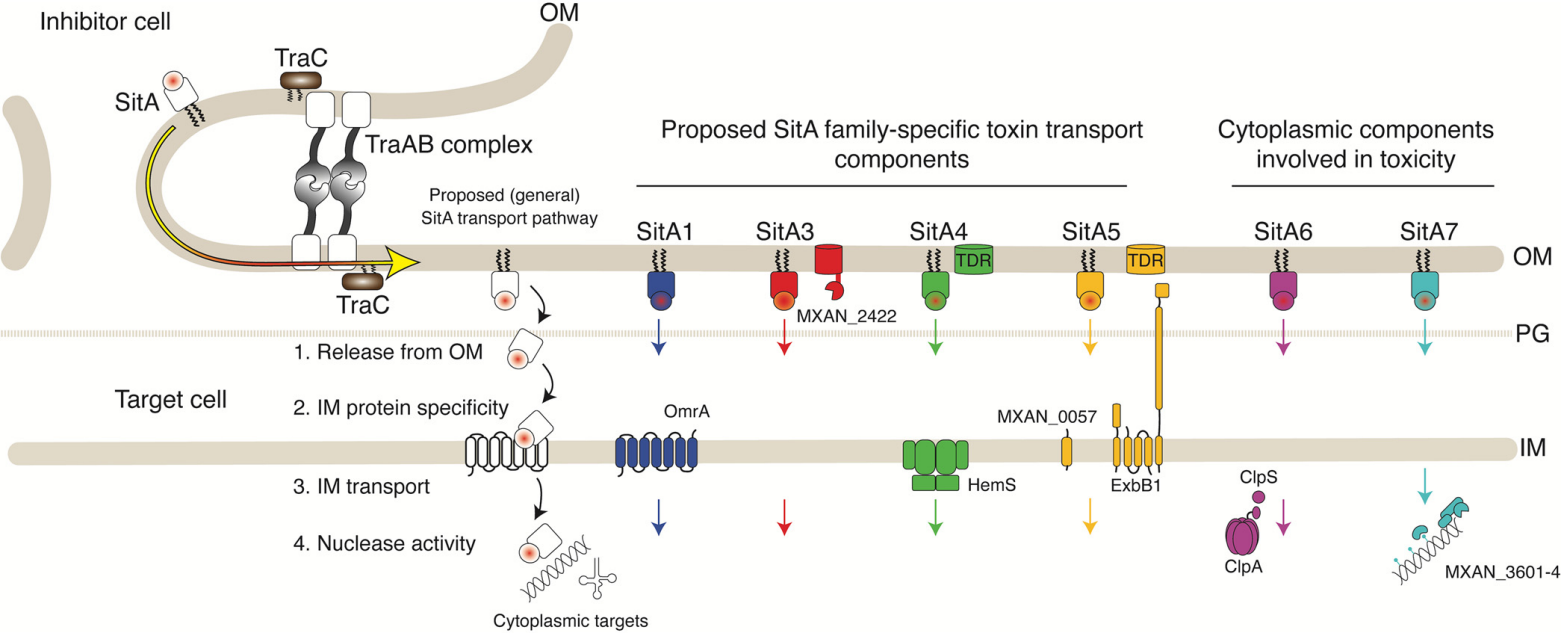
Working model of cytoplasmic entry by SitA toxins based on resistance phenotypes. TraAB receptors catalyze OM fusion between cells. SitA lipoproteins then laterally diffuse into the OM from one cell to another (yellow arrow). Each SitA family then enters the cytoplasm through unique pathways determined by its ED (squares). Proteins identified in this study are indicated. See Fig. 2C for gene neighborhood details that are depicted in the SitA4 and -5 pathways at the top. TraC is an accessory factor involved in OME. TDR, TonB-dependent receptor. See the text for details.

Bacteriocins and other toxins used in microbial warfare must reach the appropriate cellular compartment to act. In Gram-negative bacteria, toxins must cross two membranes to inhibit cytoplasmic targets and typically exploit host factors to do so (15, 24, 25). During OME, lipoprotein toxins are delivered to the inner leaflet of the OM (37, 38), but how they reach the cytoplasm of target cells was unknown. To address this question, we isolated resistant mutants, hypothesized to block cytoplasmic entry, for each of the six SitA families. Consistent with our idea, the majority of resistance loci contained proteins predicted to reside in the cell envelope (Fig. 7). Importantly, we also show that resistance phenotypes are specific to each SitA family. Additionally, we show that SitA toxins are modular, whereby their EDs were functionally swapped between families, and this domain determined the specificity of resistance. Based on these results, we propose a working model where each SitA family reaches the cytoplasm by distinct pathways determined by its ED (Fig. 7). In the case of members of the SitA3 family, we further showed that their EDs are divergent and that their resistance profiles and, hence, entry pathways were apparently different. Other SitA families may similarly have divergent entry pathways.

The ability of SitA toxins to enter the cytoplasm by discrete pathways ensures that random host mutations do not block the delivery of the entire payload. Since myxobacterial genomes contain all six families, where suites of >35 *sitAI* alleles are commonly found (14), target cells cannot easily escape poisoning by this resistance mechanism. In contrast, mutations in the *traAB* operon abolish the toxicity and delivery of all SitA toxins. Although such mutations readily arise under laboratory conditions when strains compete (29), they are not found in wild isolates, although certain myxobacterial species lack *traAB* and, consequently, *sitAI* loci (10, 13, 14). As previously described (12), these lethal selective forces also explain the high degree of polymorphisms and, hence, selectivity in recognition found within TraA receptors, which minimizes intoxication by distant kin. Additionally, these observations, combined with the fact that OME is a mutual decision between compatible TraA strains, unlike unidirectional microbial weapons like the T6SS, indicate that there are strong selective forces for retaining OME function. These fitness benefits likely pertain to cooperative behaviors exhibited by these social multicellular bacteria (39, 40).

Before SitA lipoproteins enter the cytoplasm, they must be released from the OM (Fig. 7). One plausible mechanism is by proteolysis, whereby the N-terminal lipid anchor is cleaved. Consistent with this model, SitA proteins contain intrinsically disordered regions immediately following their lipobox (14), which could serve as protease cleavage sites. Below, we discuss specific models for how the identified proteins may facilitate cytoplasmic entry by SitA toxins.

### Mutations in cell envelope proteins confer resistance to SitA toxins.

Transposon insertions in *omrA* confer specific resistance to the SitA1/2 family, including SitA1^Mx^, SitA2^Mx^, and SitA1^Mf1^. OmrA contains eight predicted transmembrane helices in the IM and likely modifies phospholipids by covalently attaching amino acids (29, 41). Since OmrA function and localization are not related to the activities of the SitA1/2 nucleases, we postulate that these toxins exploit the OmrA protein to cross the IM (Fig. 7). In an alternative model, the lack of phospholipid modification in an OmrA mutant could block the ability of SitA1/2 toxins to cross the IM (29).

A mutation in *hemS* (MXAN_1318) conferred full resistance to SitA4^Mf1^ and SitA4^Mf2^. Residing in the *hemS* gene cluster is an envelope-spanning TonB-dependent iron acquisition system (Fig. 2C), which is homologous to the Cir system of E. coli (42). Of relevance, the Cir complex is exploited by colicin Ia to enter target cells, from which it is named (colicin I receptor) (43). HemS is a cytoplasmic protein associated with an ABC transporter permease, an ATPase that shuttles iron across the IM, where HemS is thought to cleave the siderophore to release iron (42). Although *hemS* was a hit in our screen, this mutation likely causes a polar effect on the operon (Fig. 2C). In the case of colicin Ia, the toxin binds the OM via the TonB-dependent receptor Cir (also known as HmuR/CfrA, homologous to MXAN_1316), and energy derived from TonB, ExbB, and ExbD unwinds the colicin and pulls it through the Cir β-barrel (15). Colicin Ia then spontaneously assembles in the IM as a pore, which causes toxicity. SitA4 is not related to colicin Ia and instead is delivered to the inner leaflet of the OM following OME; however, the target of this nuclease is in the cytoplasm. Based on the resistance phenotype, SitA4 may exploit the Hem complex in the IM to reach the cytoplasm (Fig. 7). Additionally, there are three candidate *tonB* genes proposed to function in the M. xanthus Hem iron pathway, but they are located elsewhere in the genome (42). Future work needs to test if mutations in these genes, and ExbBD (TolQR) homologs, result in a SitA4 resistance phenotype. If so, these factors may play a role in releasing SitA4 from the OM.

Colicins exploit a small number of OM receptors and associated pathways involved in essential nutrient uptake (15). In the canonical pathway, nutrients are taken up by a TonB-dependent β-barrel OM transporter that requires interaction with the periplasm-spanning IM protein TonB, which, together with ExbBD, provides the energy for uptake (15). In fact, homologs of ExbB/D and TonB, TolQRA, respectively, were so named because mutations in these genes render tolerance to colicins, where these systems are exploited for energized colicin cell entry (15). Interestingly, we found that a mutation to the ExbB1 protein (MXAN_1448) confers full resistance to SitA5^Mf1^. MXAN_1448 is in an apparent operon located between a *tonB* gene and two *exbD* genes (Fig. 2C) and is one of eight predicted *exbB*-*tolQ* genes in M. xanthus. This operon is also adjacent to a gene that encodes a TonB-dependent transporter (MXAN_1450, also known as *oar*) (Fig. 2C) predicted to transport carbohydrates, along with other functions (44). We suggest that SitA5 exploits the ExbB1 IM protein to facilitate cytoplasmic entry (Fig. 7). Interestingly, when the ExbB1 mutant was tested against another SitA5 inhibitor, expressing the *sitA5*^Mf2^ allele, we found only partial resistance. In this regard, the members of the SitA5 family contain the most diverse sequences in their EDs. Therefore, similar to the SitA3 family, some SitA5 proteins may exploit different (e.g., homologous ExbB proteins) or multiple pathways for cytoplasmic entry. Finally, whether other members of the ExbB1 pathway (Fig. 7) are involved in SitA5 import, including release from the OM, remains to be determined.

A *mariner* insertion in MXAN_0057 conferred partial resistance to SitA5. This small open reading frame (ORF) contains a single transmembrane helix predicted to reside in the IM. Therefore, MXAN_0057 could similarly be hijacked for toxin cytoplasmic entry, possibly in conjunction with ExbB1.

A mutation to MXAN_2422 elicits strong but partial resistance to SitAI3^Mf1^. This protein has a predicted architecture that includes signal peptide (SP), peptidase, TolB-like β-propeller, and OM β-barrel domains (see Fig. S3 in the supplemental material). Therefore, we suggest that this protein could be involved in the release of SitA3 from the OM or in the maturation of the toxin for cytoplasmic entry.

### Mutations in cytoplasmic proteins confer resistance to SitA6 and SitA7.

Mutations in the *clpA* and *clpS* genes confer resistance to SitA6. ClpA is a well-conserved ATP-dependent unfoldase that typically partners with the ClpP protease and the ClpS adaptor protein to degrade protein substrates (45). M. xanthus contains many Clp family proteins, suggesting that they each target different substrates. Interestingly, in E. coli, mutations in the *clpAP* genes were found to confer resistance to antagonism mediated by the T6SS (46). These findings demonstrate a role of the Clp proteins in rendering target cells susceptible to toxins. The role of Clp proteins mediating susceptibility may be in processing toxins or in processing a host protein(s) required for toxin entry.

A mutation in MXAN_3602 confers partial resistance to SitA7. This gene contains a Z1 domain (E = 4.8^−117^), which is a putative endonuclease and is often associated with DNA helicase domains. Additionally, this gene resides in an apparent operon that codes for proteins with DNA binding domains and contains a downstream gene (MXAN_3604) that is homologous to an abortive-infection resistance protein (AIRP) gene (PF10592) (100.0% HHpred probability; E = 1.9^−50^) and shows similarity to *dndB* (91.34% HHpred probability) (47). AIRPs are associated with restriction-modification operons (48), and DndB functions with other Dnd proteins to phosphorothioate the DNA backbone (49). This operon contains a second apparent nuclease (MXAN_3603), and we therefore hypothesize that these genes constitute a restriction-modification-like system. Like other SitA toxins, SitA7 contains a predicted DNase, and in this case, SitA7 may recognize chromosomal DNA that is modified. In this scheme, mutations to the MXAN_3602 gene cluster could alter DNA modification and reduce the affinity of the SitA7 DNase for its substrate. An alternative explanation is that SitA7 causes double-strand breaks that are more toxic when the MXAN_3602 gene cluster, including its nuclease, is functional. Consistent with this, the MXAN_3602 insertion mutant provides only partial resistance.

For SitA6 and SitA7, we did not find cell envelope proteins potentially involved in cytoplasmic entry. There are a number of reasons that could explain this result. First, there may be redundancy in some SitA entry pathways, making it difficult to find mutants. Alternatively, some proteins may be essential for cell viability, thus precluding mutant isolation. Theoretically, our screen reached saturation. The number of mutants required to saturate the screen with a 99% probability is 34,325 (50). For comparison, we screened an estimated 315,000 transposon mutants against SitA6 and SitA7 inhibitor strains. However, the fact that TraC mutants were not discovered using selection against SitAI3 or SitAI5 inhibitors, and many resistant mutants were not discovered until saturation was long past, suggests that our screen had limitations in identifying certain types of mutants. One consideration here is that partially resistant mutants can be killed before they escape the mixed cultures. Additionally, any mutations that result in a motility defect or other fitness defects will not be identified.

### TraC plays a role in OME.

Mutation in *traC* confers partial or full resistance to all SitA families. Importantly, *traC* mutations in the nonmotile inhibitor relieve swarm inhibition to an extent similar to that when the mutation is in the target cell. This demonstrates that TraC is not involved in the cytoplasmic entry of toxins but instead plays a role in OME. In support of this, in stimulation assays, *traC* mutations in donor or recipient cells were also defective in the transfer of lipoproteins required for the rescue of gliding motility defects. However, since these defects are partial relative to TraA mutants, this suggests that TraC plays an accessory function with TraAB in OME. Consistent with this, the TraC lipoprotein contains TPR protein-protein binding domains and localizes to the cell envelope. Additionally, in the absence of TraC, the levels of TraAB are elevated, while the overexpression of TraC reduces their levels. Therefore, since OME activity is severely impaired in a Δ*traC* background, yet TraAB protein levels are elevated, this suggests that TraC plays a direct role in TraAB function or a role in processing them. In the former scenario, TraC could facilitate TraAB turnover following an OME fusion event. This might be required because membrane fusion requires energy, and in the OM, such energy could be derived from irreversible conformational changes in TraAB. In contrast, in the latter scenario, TraC may be required for efficient posttranslational processing, folding, and/or secretion of TraAB to the cell surface (51). Consistent with this, the top BLAST hit for TraC is the PEP_CTERM TPR lipoprotein (TIGR02917), thought to be involved in anchoring proteins to the cell surface with C-terminal tags (52). This homology is intriguing since TraA contains an analogous MYXO-CTERM motif at its C-terminal end (51). In either of these scenarios, this lack of OME activity in a Δ*traC* mutant, while TraAB levels are actually elevated, could be explained by a fraction of the TraAB protein pool being nonfunctional and perhaps even eliciting a dominant negative phenotype. Finally, we note that when *sitAI* operons contain an upstream accessory *sitB* gene, the resistance profile of *traC* mutants is partly overcome (Fig. 2B). Based on our previous work (12), this can be explained because SitB proteins increase the OME delivery efficiency of cognate SitA toxins.

## MATERIALS AND METHODS

### Bacterial strains and growth conditions.

Bacterial strains used in this study are listed in Table S1A in the supplemental material. M. xanthus was routinely cultured in the dark at 33°C with continuous shaking in CTT medium (1% Casitone, 1 mM KH_2_PO_4_, 8 mM MgSO_4_, 10 mM Tris-HCl [pH 7.6]) supplemented with kanamycin (50 μg/ml), oxytetracycline (10 μg/ml), zeocin (100 μg/ml), or isopropyl-β-d-thiogalactopyranoside (IPTG) (2 mM), as needed. For all assays, strains were grown to the logarithmic growth phase, washed, and resuspended to the appropriate density in TPM buffer (10 mM Tris [pH 7.6], 1 mM KH_2_PO_4_, and 8 mM MgSO_4_). For stimulation assays, CTT agar with 0.5% Casitone (1/2 CTT) and 2 mM CaCl_2_ was used. E. coli strains were cultured at 37°C with shaking in LB medium. For plates, 1.5% (wt/vol) agar was added.

### Genetic screen.

To avoid multiple insertions in a cell, small amounts (1 μg) of pMini-*Himar*-*lacZ* DNA were electroporated into strain DW2290. This strain contained a second ectopic copy of *traAB* to reduce the likelihood of selecting *traA* or *traB* mutants. After electroporation, the cells recovered by shaking at 33°C for 4 h (doubling time of ∼4.5 h). Recovered cultures were pelleted, divided, and spread onto 8 to 24 separate plates containing CTT agar with kanamycin (CTT Km agar). After 2 to 3 days, each plate was harvested separately into CTT Km medium and adjusted to 7.5 × 10^9^ CFU/ml. Each sample represented a separate pool containing 500 to 2,000 independent transposon mutants. Nonmotile inhibitor strains (Table S1A) were grown to mid-log phase and also adjusted to 3 × 10^9^ CFU/ml. Inhibitor cultures were then mixed with each pool of motile mutants at a 1:5 volume ratio, with the exception of the SitA6 inhibitor, which was mixed at a 5:1 ratio due to the weak activity of this toxin. These mutant libraries were screened against six different inhibitor strains in parallel that underwent 4 to 15 rounds of screening depending on the frequency of mutant isolation (Fig. S1).

In these screens, 20 μl of each mixture was spotted onto 1/2 CTT agar plates containing 2 mM CaCl_2_ and 1 mM IPTG and incubated at 33°C. Escape flares were monitored for up to 7 days and represented candidate resistant mutants, and they were toothpicked and streaked onto fresh plates to isolate single colonies. Cultures from single colonies were then subjected to swarm inhibition assays against the inhibitor that they were originally isolated against. Positive isolates were saved. To identify the site of the transposon insertion, genomic DNA was harvested and digested with SacII (New England BioLabs). The DNA was diluted, ligated with T4 DNA ligase, and transformed into DH5α *pir*^+^. Plasmid DNA was isolated from transformants and sequenced with a transposon-specific primer to identify the insertion site, as described previously (29).

To backcross the mutations into WT M. xanthus and nonmotile variants, 1 μg of genomic DNA from the original mutants was used for transformation. Transformants were recovered for 5 h in CTT medium and plated onto CTT Km medium. Transformants were then tested for the appropriate phenotype, and strain construction was verified by again cloning and sequencing the minitransposon insertion site.

### Cloning and strain construction.

Plasmids and primers used in this study are listed in Tables S1B and C, respectively. Four different chimeric SitAI constructs were made by swapping C-terminal toxin domains and cognate immunity domains between two different families (Fig. 3A). Briefly, Gibson cloning (New England BioLabs) (53) was used to ligate PCR amplicons of N-terminal EDs and C-terminal toxin immunity domains into a linearized (XbaI-KpnI) pMR3487 vector that contains an IPTG-inducible promoter and a Tc^r^ cassette (54). To construct a fusion reporter, the *traC* and *msfGFP* genes were PCR amplified and ligated into pCR-XL (EcoRI-HindIII) by Gibson assembly. Plasmids were transformed and selected in the respective M. xanthus strains. An in-frame deletion was made by PCR amplification of upstream and downstream DNA fragments of *traC* that were cloned into pBJ114 (HindIII and EcoRI) by Gibson assembly. This construct was transformed into M. xanthus and selected for integration by Km^r^. Approximately 20 transformants were pooled and grown overnight in liquid culture without selection. This culture was then back diluted in CTT medium plus 1% galactose and grown for 4 h, followed by plating onto CTT medium plus 2% galactose to counterselect for recombination and plasmid loss. Galactose-resistant cells were checked by colony PCR to identify *traC* deletion clones. All plasmids were verified by PCR, restriction digestion, and DNA sequencing before transformation into M. xanthus. Plasmids were integrated into the M. xanthus genomes by site-specific recombination at the Mx8 or Mx9 attachment sites or by homologous recombination.

### Microbiology assays.

Stimulation assays were done as described previously (9). Briefly, cells were grown to mid-log phase, harvested by centrifugation, and resuspended in TPM buffer to a calculated density of ∼2.5 × 10^9^ cells/ml. Nonmotile donor strains were mixed 1:1 with a nonmotile recipient, and 5 μl was spotted onto agar plates. Colony edges were imaged following incubation overnight at 33°C with an Olympus SZX10 stereomicroscope coupled to a digital imaging system. For swarm inhibition assays, strains were mixed 1:1, and colonies were imaged after 48 h using a Nikon E800 phase-contrast microscope with a 10× lens objective (12). For colony merger assays, competing strains were grown to mid-log phase, harvested, and resuspended in TPM buffer to a cell density of 3 × 10^9^ cells/ml. Five microliters of cells was spotted onto 1/2 CTT agar supplemented with 2 mM CaCl_2_ and IPTG. After the first spot dried, 5 μl of the competing strain was spotted next to it, with a spacing of ∼2 mm. Plates were incubated in a humid chamber in a 33°C incubator for 2 days, followed by imaging with a stereomicroscope.

### Target strain CFU.

Killing assays were set up similarly to the swarm inhibition assays, using 1:1 mixtures of the target and inhibitor strains at 7.5 × 10^9^ CFU/ml. At various times after spotting, mixed colonies were harvested and resuspended in 1 ml CTT medium. Cells were then serially diluted in CTT medium containing companion cells (Km^s^) (3 × 10^9^ CFU/ml) to facilitate plating efficiencies. Dilutions were plated onto CTT Km agar and monitored over 2 to 3 days for the formation of target colonies (nonmotile [Km^r^]). CFU were enumerated and plotted.

### Competitive index.

Competition experiments were done with modifications as described previously (12). Briefly, cells were grown to mid-log phase, harvested by centrifugation, washed in TPM buffer, and resuspended to a density of 3 × 10^9^ CFU/ml. The inhibitor and target strains were mixed 1:1 and spotted onto 1/2 CTT agar with 2 mM CaCl_2_ and 2 mM IPTG. Inocula were air dried and incubated at 33°C. At each time point, two 20-μl spots were collected, and cells were resuspended in 0.5 ml of TPM buffer. Cells were dispersed by repeated pipetting, washed, and imaged on glass slides at a 60× magnification using phase-contrast microscopy and a GFP filter set on a Nikon E800 microscope. For each sample, at least 600 total cells were counted to determine the ratio of target (GFP) to toxin-producing inhibitor (unlabeled) cells. The competitive index was plotted by calculating the relative changes in ratios from 0 to 6 or 10 and 24 h.

### Western blotting and fluorescence microscopy.

Western blot assays were done essentially as described previously (51). Primary polyclonal rabbit antibodies were anti-GFP (1:15,000 dilution; Invitrogen), anti-FLAG (1:1,500 dilution; Sigma), anti-TraA (1:25,000 dilution) (11), and anti-TraB (1:15,000 dilution) (9). For chemiluminescence detection, horseradish peroxidase (HRP)-conjugated goat anti-rabbit secondary antibody was used (1:20,000; Pierce). Blots were washed and developed using a KwikQuant imager (Kindle Biosciences LLC).

To image fluorescently tagged TraA and TraC, cells were spotted onto custom-made 0.8% agarose pads in TPM buffer and imaged using a Nikon E800 microscope with a 100× oil immersion lens. Images from GFP and mCherry filter sets were processed and overlaid using Image-Pro Plus software.

### Phylogenetic analysis.

Protein sequences for different SitA3 orthologs were aligned in MUSCLE (55) and visualized in Jalview (56) using the Clustal X default color scheme. Amino acid sequences encompassing residues 1 to 399 from MXAN_1899 (SitA3^Mx^) were used to demarcate and trim ED boundaries of all aligned orthologs. For phylogenetic analyses, a maximum likelihood phylogenetic tree was constructed in PhyML 3.0 (57). Smart Model Selection was used to select a substitution model (58) with 1,000 bootstrap replicates. The phylogenetic tree was visualized in FigTree v1.4.4 (http://tree.bio.ed.ac.uk/software/figtree/). A pairwise amino acid sequence alignment was performed for two SitA3 orthologs using default parameters in MUSCLE.

### Kill dynamics movie.

A glass slide was fitted with a custom-made incubation chamber. To do this, four Frame-Seal incubation chambers (Bio-Rad) were stacked on top of each other to create an ∼2-mm-deep chamber. Next, 300 μl of molten 0.8% agarose in 1/2 CTT medium containing 2 mM CaCl_2_ and 2 mM IPTG (∼55°C) was pipetted into the chamber, and a coverslip was placed immediately on top to create a flat surface. After removing the coverslip, 2 μl of a 3:1 ratio of a GFP-labeled target and unlabeled inhibitor cell mixture was spotted at a cell density of 3 × 10^9^ CFU/ml. Following air drying, a coverslip was placed on the agarose pad, and the mixture was incubated at 33°C for 30 min before imaging. Images were captured at 30-s intervals for ∼12 h and compiled at 10 frames/s using an Olympus IX83 inverted microscope fitted with a 60× oil immersion lens objective and an Orca-Flash4.0 LT sCMOS camera.
