# Supplementary figures and images for: Modular Lipoprotein Toxins Transferred by Outer Membrane Exchange Target Discrete Cell Entry Pathways
Source: mBio. 2021 Sep 14;12(5):e02388-21. doi: 10.1128/mBio.02388-21 (PMC8546572; doi:10.1128/mBio.02388-21)

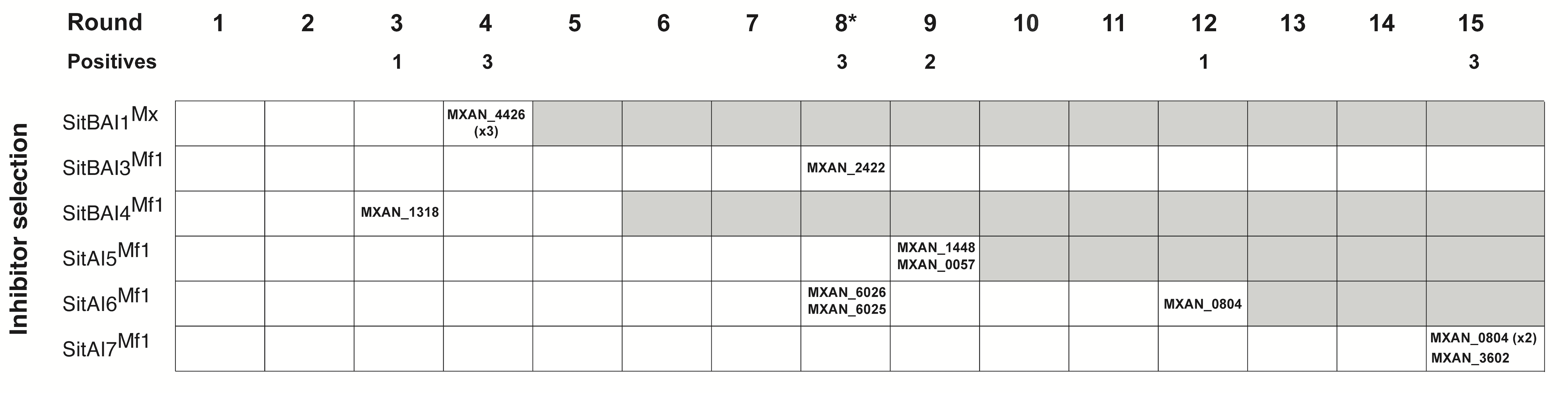

Supplement: FIG S1 [file mbio.02388-21-sf001.tif]

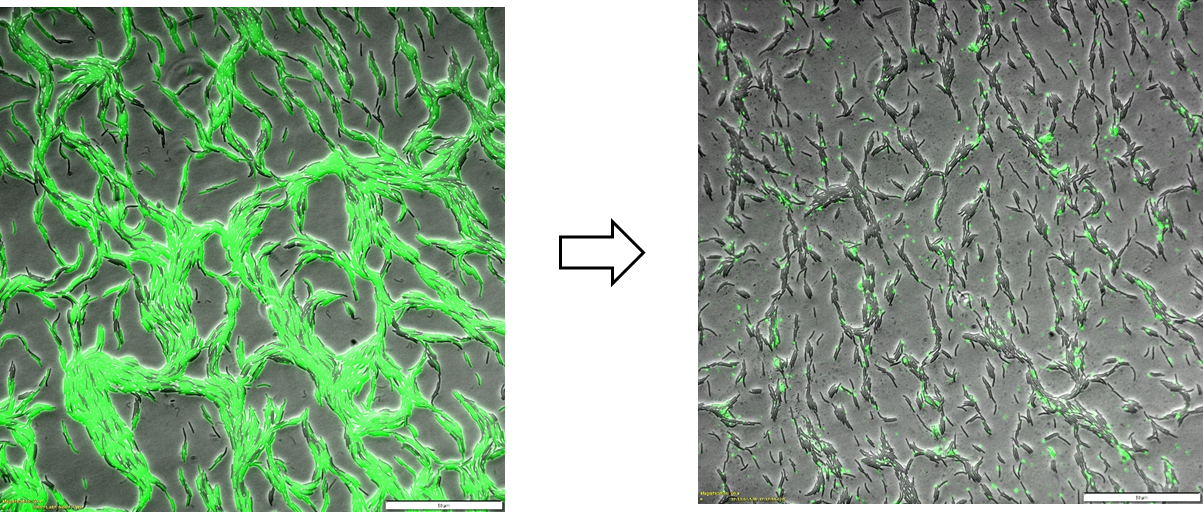

Supplement: FIG S2 [file mbio.02388-21-sf002.tif]

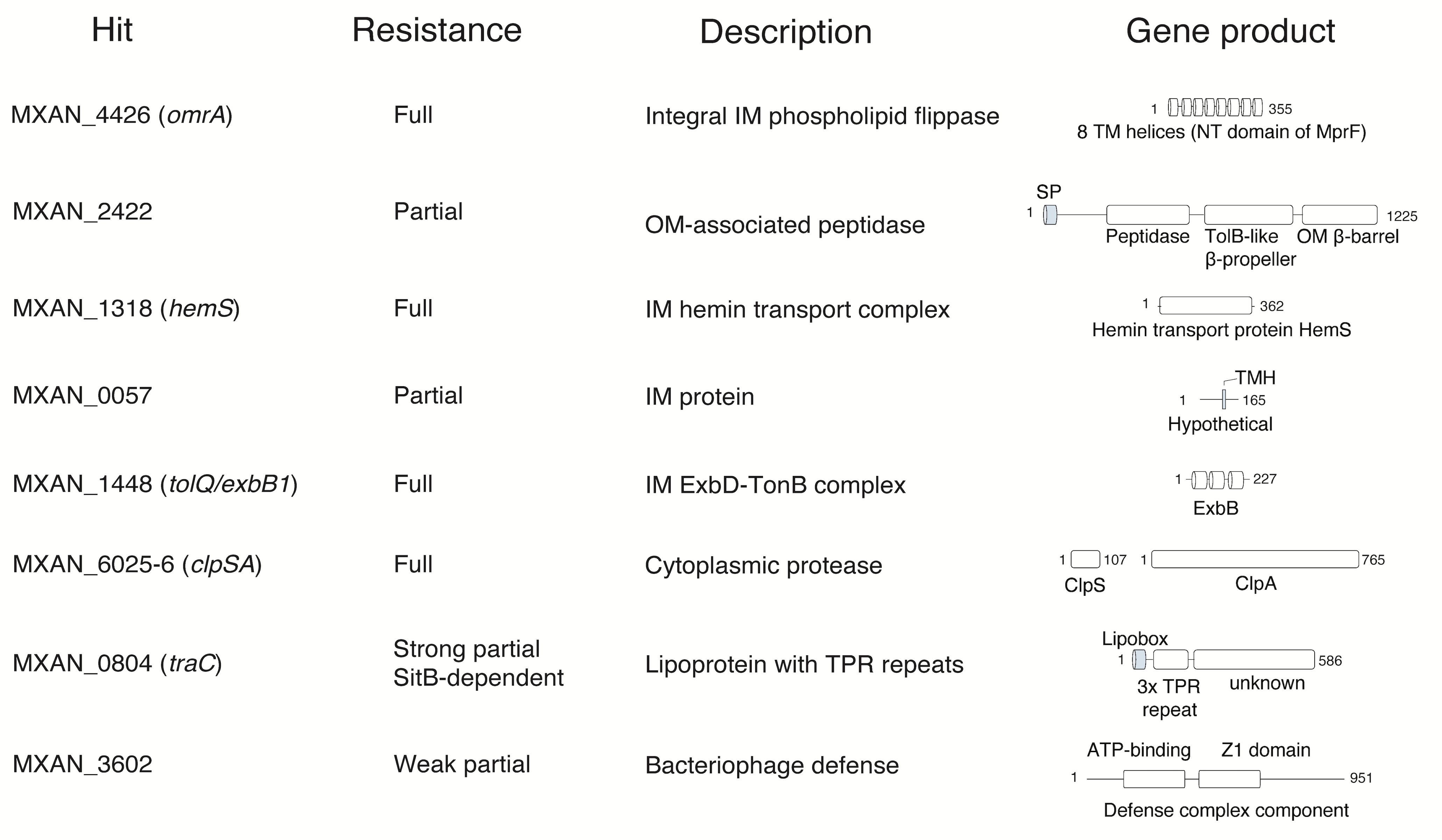

Supplement: FIG S3 [file mbio.02388-21-sf003.tif]

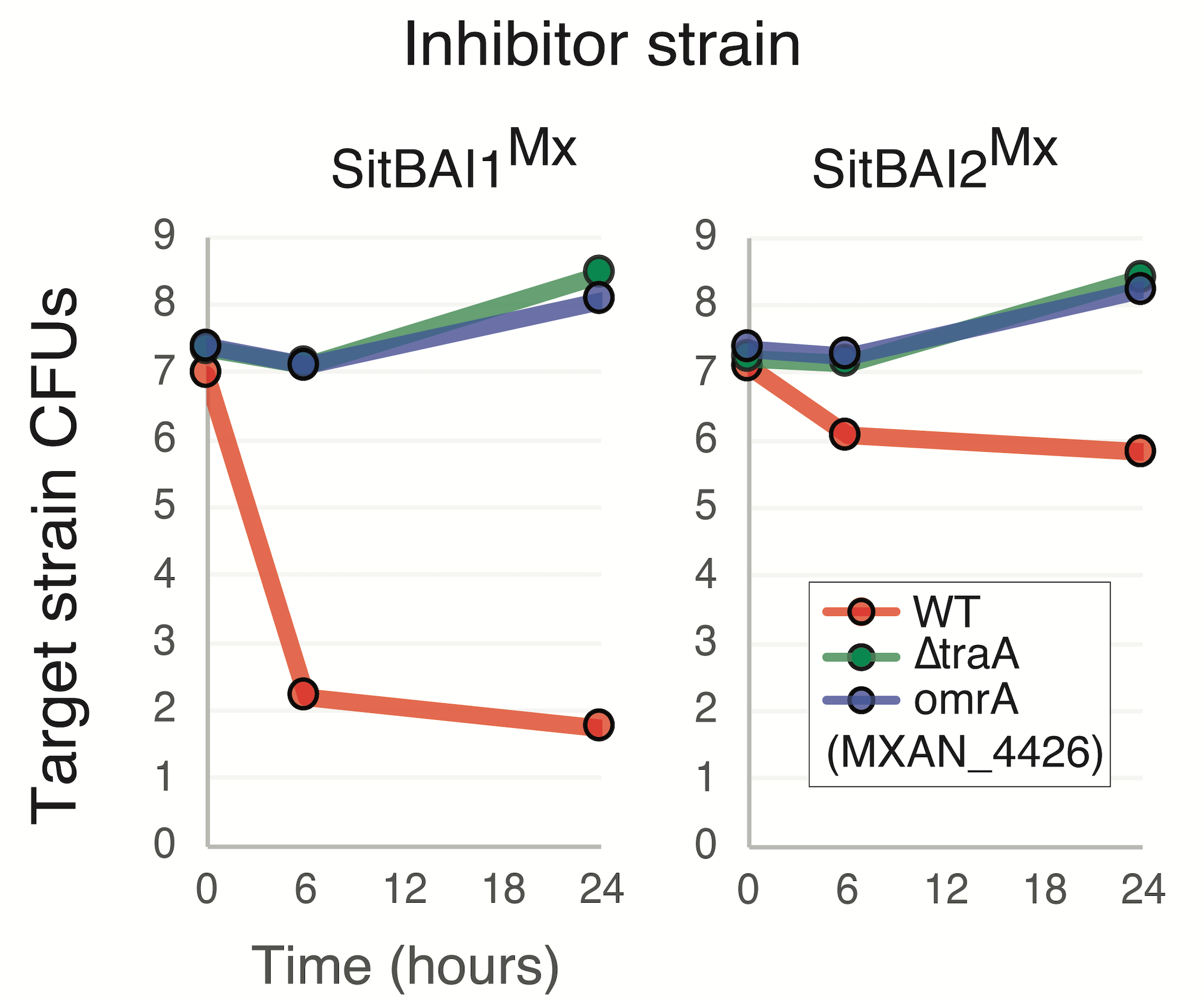

Supplement: FIG S4 [file mbio.02388-21-sf004.tif]

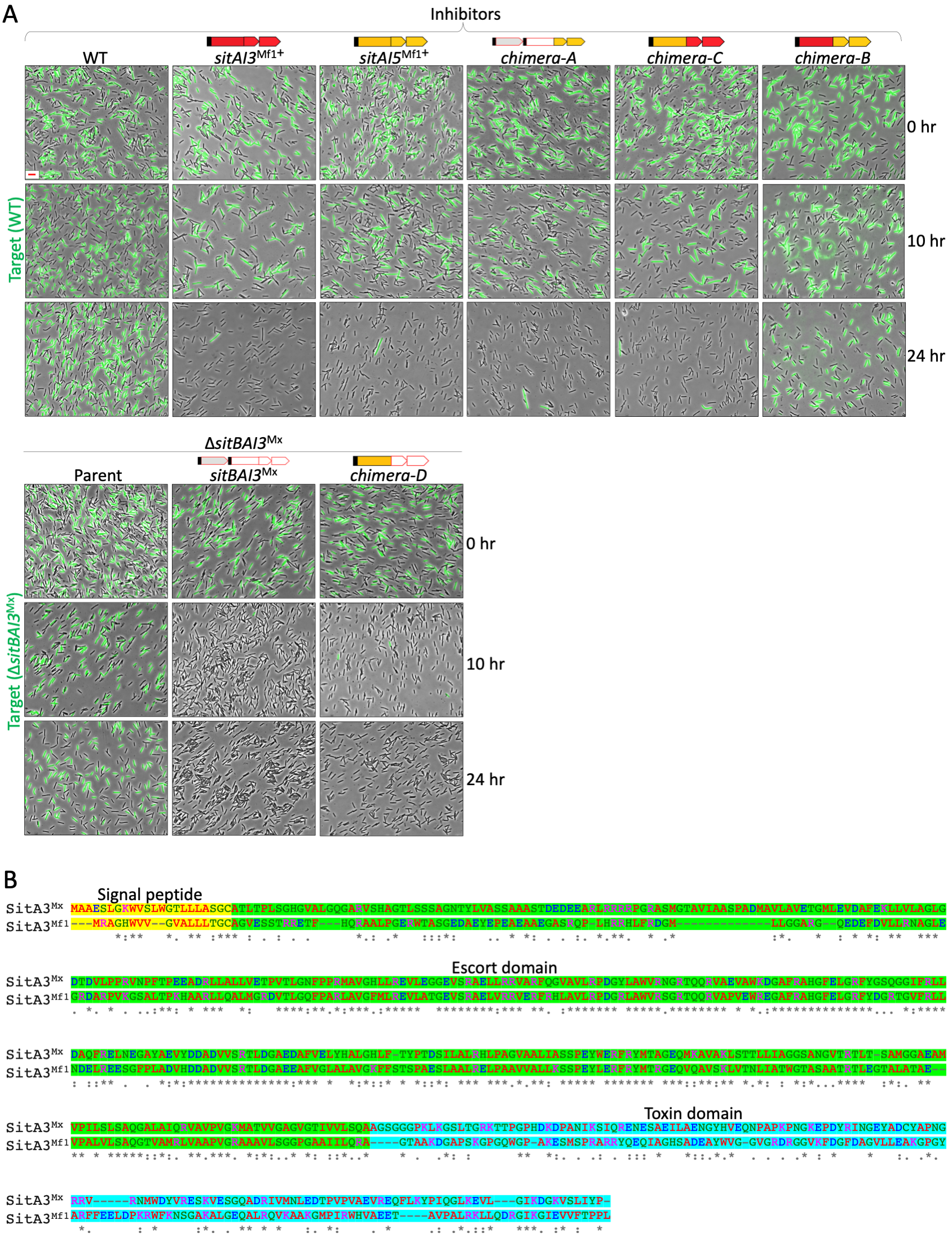

Supplement: FIG S5 [file mbio.02388-21-sf005.tiff]

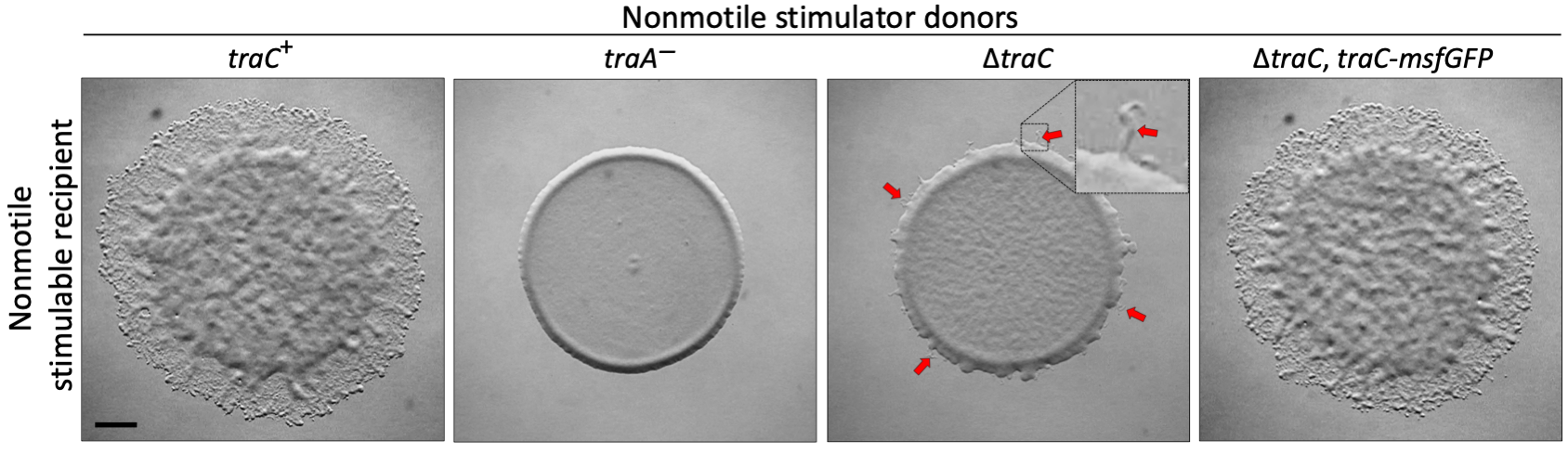

Supplement: FIG S6 [file mbio.02388-21-sf006.tiff]

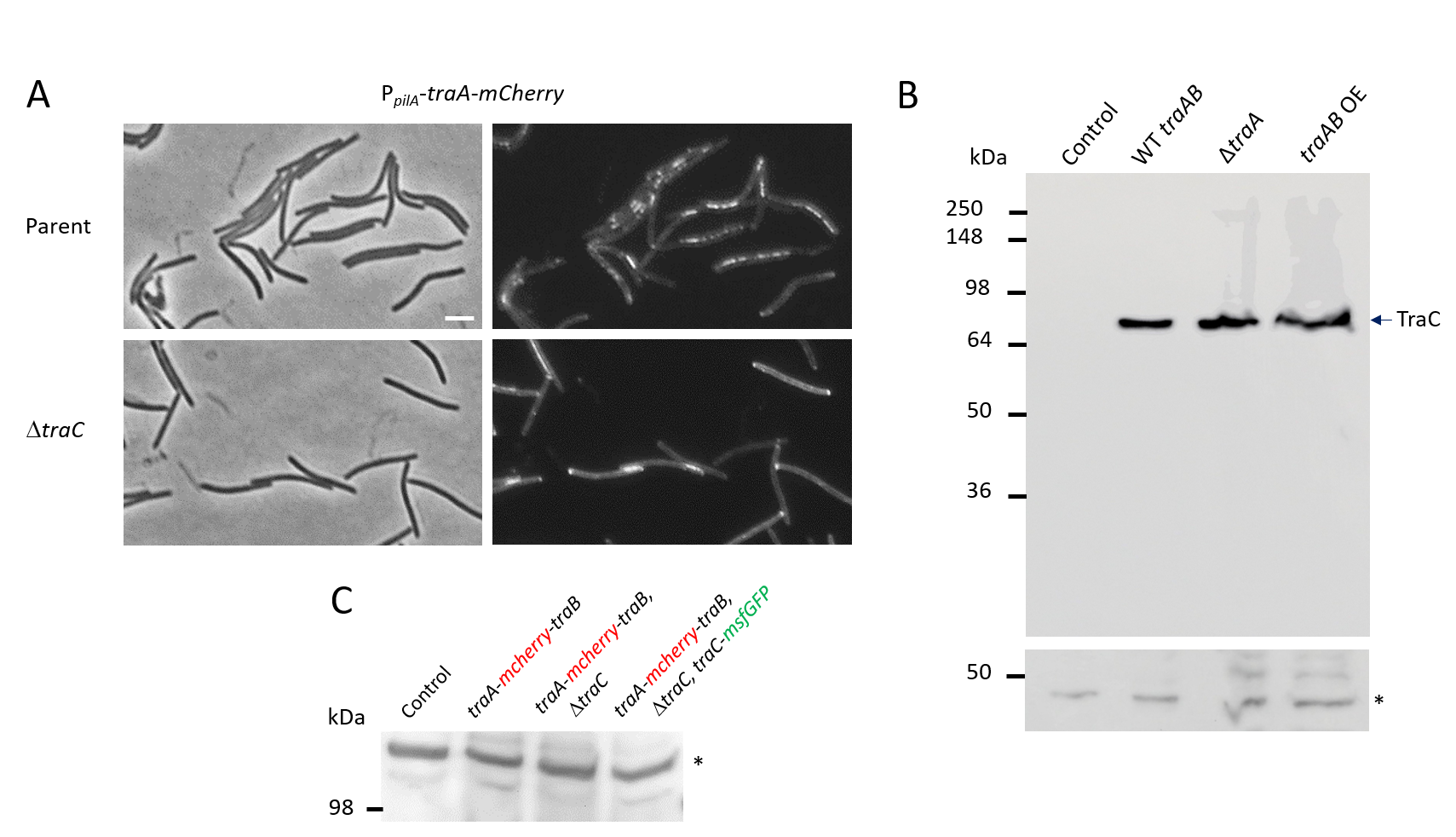

Supplement: FIG S7 [file mbio.02388-21-sf007.tif]
