# Supplementary material for: Modular Lipoprotein Toxins Transferred by Outer Membrane Exchange Target Discrete Cell Entry Pathways
Source: mBio. 2021 Sep 14;12(5):e02388-21. doi: 10.1128/mBio.02388-21 (PMC8546572; doi:10.1128/mBio.02388-21)
Supplement: TABLE S1 [file mbio.02388-21-st001.docx]

**Table S1A** Strains used in this study

| Strains | Genotype | Experimental use (figures) | Source |
| --- | --- | --- | --- |
| TOP10 | *E. coli* cloning strain | Cloning | Invitrogen |
| DH5α *pir*+ | *E. coli* strain for *oriR6K* replication | Cloning *Himar*-*lacZ oriR6K* inserts | Lab collection |
| DK1622 | Wild type *M. xanthus* | 3B, 3C, 6A, S7B | [1] |
| DK6204 | DK1622 ∆*mglBA* (A^−^S^−^; nonmotile) | 3C, 3D, 6B, S5A, S6 | [2] |
| DW2290 | DK1622 P_IPTG_-*traAB* ^(ATG)^, Tc^r^ (aka PC_Mx_321) | Screening strain; 1C | Lab collection |
| DW2459 | DK6204 P_IPTG_-*sitBAI*1^Mx^, Tc^r^ | 1C | [3] |
| DW2461 | DK6204 P_IPTG_-*sitAI3*^Mf1^, Tc^r^ | 1C, 3C, 3D, S5A | [3] |
| DW2462 | DK6204 P_IPTG_-*sitBAI4*^Mf1^, Tc^r^ | 1C | [3] |
| DW2463 | DK6204, P_IPTG_-*sitAI5*^Mf1^, Tc^r^ | 1C, 3C, 3D, S5A | [3] |
| DW2464 | DK6204 P_IPTG_-*sitAI6*^Mf1^, Tc^r^ | 1C | [3] |
| DW2465 | DK6204 P_IPTG_-*sitAI7*^Mf1^, Tc^r^ | 1C, 6A | [3] |
| DW2498 | DK1622 *omrA*::mini-*Himar*, Km^r^ | 1C | This study |
| DW2499 | DK1622 *MXAN_2422*::mini-*Himar*, Km^r^ | 1C | This study |
| DW2600 | DK1622 *hemS*::mini-*Himar*, Km^r^ | 1C | This study |
| DW2601 | DK1622 *tolQ*::mini-*Himar*, Km^r^ | 1C | This study |
| DW2602 | DK1622 *MXAN_0057*::mini-*Himar*, Km^r^ | 1C | This study |
| DW2603 | DK1622 *MXAN_0804*::mini-*Himar*, Km^r^ | 1C | This study |
| DW2604 | DK1622 *clpA*::mini-*Himar*, Km^r^ | 1C | This study |
| DW2605 | DK1622 *MXAN_3602*::mini-*Himar*, Km^r^ | 1C | This study |
| DW1409 | DK6204 *traA*::*km*^r^ | 2A, 2B, S4, S6 | [4] |
| DW2441 | DK1622 P_IPTG_-*sitAI3*^Mf1^, Tc^r^ | 2A, 2B, 3B, 4A | [3] |
| DW2445 | DK1622 P_R3/4_-*eGFP*, Km^r^ | 2A, 2B, 3D, S5A | [5] |
| DW2451 | DK1622 P_IPTG_-*sitBAI4*^Mf1^, Tc^r^ | 2A, 2B | [3] |
| DW2452 | DK1622 P_IPTG_-*sitAI4*^Mf2^, Tc^r^ | 2A, 2B | [3] |
| DW2453 | DK1622 P_IPTG_-*sitAI5*^Mf1^, Tc^r^ | 2A, 2B, 3B, 4A | [3] |
| DW2454 | DK1622 P_IPTG_-*sitAI5*^Mf2^, Tc^r^ | 2A | [3] |
| DW2455 | DK1622 P_IPTG_-*sitAI6*^Mf1^, Tc^r^ | 2A, 2B | [3] |
| DW2456 | DK1622 P_IPTG_-*sitAI6*^pMf1^, Tc^r^ | 2A | [3] |
| DW2457 | DK1622 P_IPTG_-*sitAI7*^Mf1^, Tc^r^ | 2A, 2B | [3] |
| DW2607 | DK6204 *omrA*::mini-*Himar*, Km^r^ | 2A, 2B, S4 | This study |
| DW2608 | DK6204 *MXAN_2422*::mini-*Himar*, Km^r^ | 2A, 2B | This study |
| DW2609 | DK6204 *hemS*::mini-*Himar*, Km^r^ | 2A, 2B | This study |
| DW2610 | DK6204 *tolQ*::mini-*Himar*, Km^r^ | 2A, 2B | This study |
| DW2611 | DK6204 *MXAN_0057*::mini-*Himar*, Km^r^ | 2A, 2B | This study |
| DW2612 | DK6204 *MXAN_0804*::mini-*Himar*, Km^r^ | 2A, 2B | This study |
| DW2613 | DK6204 *clpA*::mini-*Himar*, Km^r^ | 2A, 2B | This study |
| DW2614 | DK6204 *MXAN_3602*:: mini-*Himar*, Km^r^ | 2A, 2B | This study |
| DW2415 | DK1622 P_IPTG_-*sitBAI1*^Mx^, Tc^r^ | 2A, 2B, S4 | [5] |
| DW2617 | DK6204 P_R3/4_-*eGFP*, Km^r^ | 2A, 2B, S4 | This study |
| DW2438 | DK1622 ∆s*itBAI3*^Mx^ P_IPTG_-*sitBAI3*^Mx^, Tc^r^ | 2B, 2D, 3B, 3D, 5B, S2, S5A, movie S1 | [5] |
| DW2532 | DK1622 P_IPTG_-*sitA3*^Mf1^-*AI5*^Mf1^, Tc^r^ | 2B, 3B | This study |
| DW2435 | DK1622 ∆s*itBAI3*^Mx^ | 3B, 3D, S5A | [5] |
| DW2524 | DK1622 ∆*sitBAI3*^Mx^ P_IPTG_-*sitA5*^Mf1^*-AI3*^Mx^, Tc^r^ | 3B, 3D, 4B, S5A | This study |
| DW2526 | DK1622 P_IPTG_-*sitBA3*^Mx^*-AI5*^Mf1^, Tc^r^ | 3B, 4A | This study |
| DW2527 | DK1622 P_IPTG_-*sitA5*^Mf1^*-AI3*^Mf1^, Tc^r^ | 3B, 4A | This study |
| DW2525 | DK6204 P_IPTG_-*sitA5*^Mf1^*-AI3*^Mf1^, Tc^r^ | 3C, 3D, S5A | This study |
| DW2523 | DK6204 P_IPTG_-*sitBA3*^Mx^*-AI5*^Mf1^, Tc^r^ | 3C, 3D, S5A | This study |
| DW2533 | DK6204 P_IPTG_-*sitA3*^Mf1^-*AI5*^Mf1^, Tc^r^ | 3C, 3D, S5A | This study |
| DW2615 | DK1622 ∆*sitBAI3*^Mx^ P_R3/4_-*eGFP*, Km^r^ | 3D, 5B, S2, S5A, movie S1 | This study |
| DW2528 | DK1622 *tolB*:: *Himar*, P_IPTG_-*sfGFP*, Km^r^ Tc^r^ | 4A | This study |
| DW2529 | DK1622 *tolQ*:: *Himar*, P_IPTG_-*sfGFP,* Km^r^ Tc^r^ | 4A | This study |
| DW2530 | DK1622 ∆*sitBAI3*^Mx^ *tolQ*:: *Himar*, Km^r^ | 4B | This study |
| DW2534 | DK1622 ∆*sitBAI3*^Mx^ P_IPTG_-*tdTomato* *tolB*:: *Himar*, Km^r^ Tc^r^ | 4B | This study |
| DW2535 | DK1622 ∆*sitBAI3*^Mx^ P_IPTG_-*sitAI5*^Mf1^, Tc^r^ | 4B | This study |
| DW2439 | DK1622 ∆*sitBAI3*^Mx^ P_IPTG_*-sitAI3*^Mf1^, Tc^r^ | 4B, 5B | [5] |
| DW2531 | DK1622 ∆*sitBAI3*^Mx^ *tolB*:: *Himar*, Km^r^ | 5B | This study |
| DW1480 | DK1622 ∆*traA* | 6A | [6] |
| DW2619 | DK6204, P_IPTG_-*sitAI7*^Mf1^, *traC*:: mini-*Himar*, Km^r^ Tc^r^ | 6A | This study |
| DW2620 | DK1622 ∆*traC* | 6A | This study |
| DW1466 | DK1622 ∆*cglC* ∆*tgl* | 6B, S6 | [4] |
| DW2621 | DK1622 ∆*cglC* ∆*tgl* ∆*traC* | 6B | This study |
| DW2616 | DK6204 ∆*traC* | 6B, S6 | This study |
| DW2522 | DW1483 ∆*traC* P*_pilA_*-*traA*-*mCherry-traB* P*_pilA_*-*traC*-*msfGFP,* Zeo^r^ Tc^r^ Km^r^ | 6C, 6D | This study |
| DW1483 | DK8601 ∆*traAB* | 6D | [7] |
| DW2279 | DK1483 P*_pilA_-traA-mCherry-traB*, Km^r^ | 6D | [8] |
| DW2618 | DW1483 ∆*traC* P*_pilA_*-*traA*-*mCherry-traB,* Km^r^ Tc^r^ | 6D | This study |
| DW2416 | DK1622 P_IPTG_-*sitBAI2*^Mx^, Tc^r^ | S4 | [5] |
| DW2536 | DW2616 P*_pilA_*-*traC*-*msfGFP*, Zeo^r^ | S6 | This study |
| DW2800 | DK1622 *traC*^ATG^-FLAG, Zeo^r^ | S7B | This study |
| DW2801 | DK1622 P_IPTG_-*traAB, traC*^ATG^-FLAG, Zeo^r^ | S7B | This study |
| DW2802 | DK1622 ∆*traA, traC*^ATG^*-*FLAG, Zeo^r^ | S7B | This study |

**Table S1B** Plasmids used in this study

| Plasmids | Relevant features | Source |
| --- | --- | --- |
| pSWU19 | *M. xanthus* cloning vector, Km^r^ | [9] |
| pDP22 | pSWU19 Mx8 *attP/int* cassette, P*_pilA_*, Km^r^ | [10] |
| pMR3487 | IPTG-inducible promoter, Tc^r^ | [11] |
| pCR-XL-TOPO | Cloning vector, Km^r^ Zeo^r^ | Invitrogen |
| pCR-XL-Mx9 | pCR-XL-TOPO Δ*kan* Mx9 *attP/int* cassette, Zeo^r^ | Lab collection |
| pMini-*Himar*-*lacZ* | *Mariner* family derived transposon with *lacZ* *oriR6K*, Km^r^ | Heidi Kaplan; [12] |
| pPC44 | pSWU19 P*_pilA_-traA-mCherry-traB,* Km^r^ | [8] |
| pPC230 | pMR3487-*traAB*^ATG^, Tc^r^ | Lab collection |
| pCV52 | pMR3487-*sfgfp*, Tc^r^ | This study |
| pCV182 | pSWU19 P_R3/4_-*eGFP*, Km^r^ | This study |
| pCV279 | pBJ114-Δ*traC*, Km^r^ Gal^s^ | This study |
| pGS180 | pCR-XL-Mx9-P*_pilA_*-*traC*^ATG^-FLAG, Zeo^r^ | This study |
| pGS182 | pCR-XL-Mx9 P*_pilA_*-RBS_syn_-*traC*^ATG^-*msfGFP*, Zeo^r^ | This study |
| pGS185 | pMR3487-*sitBA3*^Mx^*-AI5*^Mf1^, Tc^r^ | This study |
| pGS189 | pMR3487-*sitA5*^Mf1^*-AI3*^Mx^, Tc^r^ | This study |
| pGS191 | pMR3487-*sitA5*^Mf1^*-AI3*^Mf1^, Tc^r^ | This study |
| pGS199 | pMR3487-*sitA3*^Mf1^*-AI5*^Mf1^, Tc^r^ | This study |

**Table S1C** Primers used in this study

| Primer name | Sequence (5’→ 3’) |
| --- | --- |
| SitBA3-Mx_F1 | TGAGCGGATAACAATTAAGGAGGCTCTAGAATGTCTCTGAGTCGATGT |
| SitBA3-Mx_R1 | CGCCTGACTGAGGACAAC |
| SitAI5-Mf1a_F2 | AACCATCGTTGTCCTCAGTCAGGCGATGGCGGCCAGCAGCGCG |
| SitAI5-Mf1c_R2 | GACCATGATTACGAAGGCGAGCTCGGTACCCTACTCCGGGTCAAGCGC |
| SitA5-Mf1c_F1 | TGAGCGGATAACAATTAAGGAGGCTCTAGAATGCTGCCGCGATGGTCG |
| SitA5-Mf1c_R1 | GGCCACCGCGCCGGGTGC |
| SitAI3-Mx_F2 | CGCCCTCGCACCCGGCGCGGTGGCCGCAGGAAGTGGCGGTGGA |
| SitAI3-Mx_R2 | GACCATGATTACGAAGGCGAGCTCGGTACCCTAGTCAGAACTGGTGCA |
| SitA3-Mf1_F1 | TGAGCGGATAACAATTAAGGAGGCTCTAGAATGCGTGCTGGCCATTGG |
| SitBA3-Mf1_R1 | CGCCCGCTGAAGAATGAT |
| SitAI5-Mf1b_F2 | GGCGGCCATCATTCTTCAGCGGGCGATGGCGGCCAGCAGCGCG |
| SitAI3-Mf1_F2 | CGCCCTCGCACCCGGCGCGGTGGCCGGCACCGCCGCGAAGGAC |
| SitAI3-Mf1_R2 | GACCATGATTACGAAGGCGAGCTCGGTACCCTATGGTTGGGCCTGGAG |
| 804^ATG^-GFP/FLAG _F1 | GAATACTCAAGCTATGCATCAAGCTTcgtcatgttggacgaggt |
| 804^ATG^-GFP/FLAG _R1 | TAATTCTCCTTTCTATTTCTATTCT |
| 804^ATG^-GFP/FLAG _F2 | AGAATAGAAATAGAAAGGAGAATTAATGCCTGCCGGGATGGGGG |
| 804^ATG^-GFP_R2 | ACAGTTCTTCACCTTTACTGCCGCCGCCGCCGCTCAGCCCCACCAGGCTCTC |
| 804^ATG^-GFP_F3 | GGCGGCAGTAAAGGTGAA |
| 804^ATG^-GFP_R3 | gctcggtcgactccaagggcGAATTCCTATTTGTAGAGTTCATCCAT |
| 804^ATG^-FLAG_R2 | GCTCGGTCGACTCCAAGGGCGAATTCCTACTTGTCGTCATCGTCTTTGTAGTCCAGCCCCACCAGGCTCTC |
| ∆804_upstrm-F | AACAGCTATGACCATGATTACGCCAAGCTTCACCGAAGAGAAGTTGAGCG |
| ∆804_upstrm-R | TCCGGACGATTCACTGCATCTTCGAGGAAAGCC |
| ∆804_downstrm-F | CTCGAAGATGCAGTGAATCGTCCGGAGAGCCTG |
| ∆804_downstrm-R | CGACGTTGTAAAACGACGGCCAGTGAATTCGATGTACTGGCCATTCACG |

Restriction sites underlined.

**References**

1. Dey, A., et al., *Sibling rivalry in Myxococcus xanthus is mediated by kin recognition and a polyploid prophage.* J Bacteriol, 2016. **198**(6): p. 994-1004.

2. Hartzell, P. and D. Kaiser, *Upstream gene of the mgl operon controls the level of MglA protein in Myxococcus xanthus.* J Bacteriol, 1991. **173**(23): p. 7625-35.

3. Vassallo, C.N. and D. Wall, *Self-identity barcodes encoded by six expansive polymorphic toxin families discriminate kin in myxobacteria.* Proc Natl Acad Sci U S A, 2019. **116**(49): p. 24808-24818.

4. Pathak, D.T., et al., *Cell contact-dependent outer membrane exchange in myxobacteria: genetic determinants and mechanism.* PLoS Genet, 2012. **8**(4): p. e1002626.

5. Vassallo, C.N., et al., *Infectious polymorphic toxins delivered by outer membrane exchange discriminate kin in myxobacteria.* eLife, 2017. **6**.

6. Vassallo, C., et al., *Cell rejuvenation and social behaviors promoted by LPS exchange in myxobacteria.* Proc Natl Acad Sci U S A, 2015. **112**(22): p. E2939-46.

7. Cao, P. and D. Wall, *Self-identity reprogrammed by a single residue switch in a cell surface receptor of a social bacterium.* Proc Natl Acad Sci U S A, 2017. **114**(14): p. 3732-3737.

8. Cao, P.B. and D. Wall, *Direct visualization of a molecular handshake that governs kin recognition and tissue formation in myxobacteria.* Nat Commun, 2019. **10**.

9. Wu, S.S. and D. Kaiser, *Genetic and functional evidence that type IV pili are required for social gliding motility in Myxococcus xanthus.* Mol Microbiol, 1995. **18**(3): p. 547-58.

10. Pathak, D.T., et al., *Molecular recognition by a polymorphic cell surface receptor governs cooperative behaviors in bacteria.* PLoS Genet, 2013. **9**(11): p. e1003891.

11. Iniesta, A.A., et al., *Two systems for conditional gene expression in Myxococcus xanthus inducible by isopropyl-beta-D-thiogalactopyranoside or vanillate.* J Bacteriol, 2012. **194**(21): p. 5875-85.

12. Youderian, P., et al., *Identification of genes required for adventurous gliding motility in Myxococcus xanthus with the transposable element mariner.* Mol Microbiol, 2003. **49**(2): p. 555-70.
